# Enhancing Fatigue Life and Strength of Adhesively Bonded Composite Joints: A Comprehensive Review

**DOI:** 10.3390/ma16196468

**Published:** 2023-09-28

**Authors:** Hossein Malekinejad, Ricardo J. C. Carbas, Alireza Akhavan-Safar, Eduardo A. S. Marques, Fernando Castro Sousa, Lucas F. M. da Silva

**Affiliations:** 1 Instituto de Ciência e Inovação em Engenharia Mecânica e Engenharia Industrial (INEGI), Rua Dr. Roberto Frias, 4200-465 Porto, Portugal; 2 Departamento de Engenharia Mecânica, Faculdade de Engenharia (FEUP), Universidade Do Porto, Rua Dr. Roberto Frias, 4200-465 Porto, Portugal

**Keywords:** adhesively bonded joints, composite materials, fatigue, fatigue life, geometry effects

## Abstract

Adhesive bonding is widely seen as the most optimal method for joining composite materials, bringing significant benefits over mechanical joining, such as lower weight and reduced stress concentrations. Adhesively bonded composite joints find extensive applications where cyclic fatigue loading takes place, but this might ultimately lead to crack damage and safety issues. Consequently, it has become essential to study how these structures behave under fatigue loads and identify the remaining gaps in knowledge to give insights into new possibilities. The fatigue life of adhesively bonded composite joints is influenced by various parameters, including joint configuration and material properties of adherends and adhesive. Numerous studies with varying outcomes have been documented in the literature. However, due to the multitude of influential factors, deriving conclusive insights from these studies for practical design purposes has proven to be challenging. Hence, this review aims to address this challenge by discussing different methods to enhance the fatigue performance of adhesively bonded composite joints. Additionally, it provides a comprehensive overview of the existing literature on adhesively bonded composite joints under cyclic fatigue loading, focusing on three main aspects: Adherends modification, adhesive modification, and joint configurations. Since the effect of modifying the adhesive, adherends, and joint configurations on fatigue performance has not been comprehensively studied in the literature, this review aims to fill this gap by compiling and comparing the relevant experimental data. Furthermore, this review discusses the challenges and limitations associated with the methods that can be used to monitor the initiation and propagation of fatigue cracks.

## 1. Introduction

In recent years, both academia and industry have been making an extensive effort to develop new materials and structures that are lightweight and high performing, while also considering environmental and energy concerns. Composite materials have gained significant attention as a potential constructive solution [1,2], but manufacturing complex composite structures in large dimensions poses challenges in terms of transportation, repair and maintenance, quality control, and cost optimization [3,4,5]. As a result, composite structures are often produced in multiple parts and then joined together using various joining methods [6]. Adhesively bonded joints are commonly used in composite structures such as aerostructures [7,8], wind turbines [9], automobiles [10], naval construction [11,12], and civil [13] due to their advantages. They may also be used to repair failed parts of engineering structures or replace older sections [14,15,16]. Compared to mechanical joining methods, adhesive joints have the advantage of distributing the load over a larger area, resulting in stronger bonds [17]. Adhesive bonding is also useful for bonding similar and dissimilar materials of varying thicknesses [18,19]. Considering this level of complexity and the variety of applications, adhesive systems are susceptible to various mechanical failure modes within the different loading conditions [20]. Hence, focusing on the improvement of the performance and investigating the failure modes of adhesively bonded joints in composite structures should be taken into account. 

**Figure 1:**
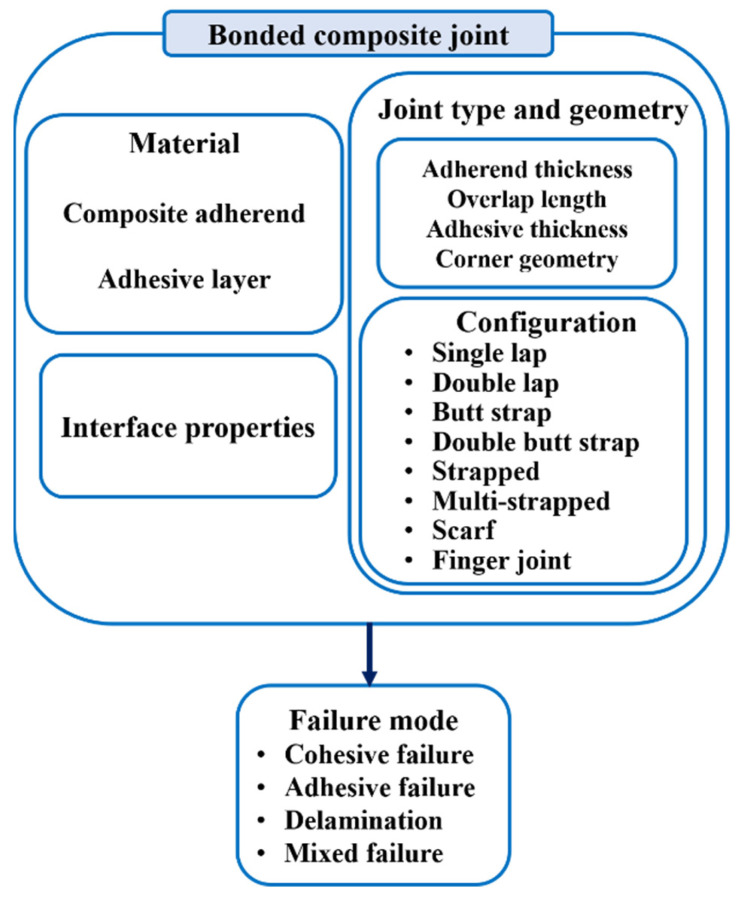
Effective parameters influencing fatigue life in adhesively bonded composite joints (adapted from [24,27]).

Although adhesively bonded composite joints have been extensively studied for their performance under static loading conditions [21,22,23], their behavior under fatigue loading has not received the same level of attention. It is unclear whether the techniques used to improve the strength of adhesively bonded joints in composite structures under static loading conditions would have the same effect on their performance under cyclic fatigue loading conditions. The use of composites as adherends can increase the complexity of fatigue failure due to the potential occurrence of delamination within the adherends [24,25,26]. Therefore, studying the fatigue behavior of bonded joints with composite adherends can present greater difficulties compared to metals. This is primarily because of the complex nature of the fatigue failure process in sophisticated structures. Moreover, studying the fatigue behavior of adhesively bonded composite joints can be a difficult task due to the multitude of influencing factors. These factors include the material properties and geometry of the adherends and the adhesive and the surface treatment method. Referring to Figure 1, which illustrates these parameters, it is evident that by modifying the material and geometry of the adhesive and adherends, both the fatigue characteristics and failure modes of adhesively bonded composite joints can be altered.

The fatigue life of a composite adhesive joint is impacted by the distribution of stresses along the adhesive and adherends, as well as their interface, which is a function of various parameters, including joint configurations, material properties of adherends and adhesive [28], the service temperature [20,29], the humidity level [30], the manufacturing process, and associated surface treatment [31]. The fatigue life can be controlled by modifying these parameters, such as hybridizing the adherends and adhesive [32,33], incorporating additive particles [34], and adjusting the configurations [35]. 

The limitations in the application of adhesive bonded joints in primary structures are caused by the uncertainty surrounding the quality of adhesive joint manufacturing, as well as the inability of current non-destructive health monitoring methods to accurately detect failures, especially when cracks are initiated by fatigue. Fatigue failures in primary structural adhesive joints can be difficult to detect in their early stages, which increases the risk of sudden and unexpected fracture. Consequently, a part of this review paper is dedicated to the methods developed for monitoring fatigue initiation and crack growth. 

The majority of applications that utilize adhesively bonded joints are exposed to high cyclic fatigue loading over their lifespan, and fatigue failure is well known to be one of the primary causes of catastrophic failure in many cases (up to 50–90%) [36]. This fact highlights the importance of studying fatigue behavior in adhesively bonded joints. This is particularly crucial, especially when designing under safe life and damage tolerance philosophies [17,37]. 

Despite the publication of a few review papers on the fatigue behavior of adhesively bonded composite joints [27,38,39], there is a lack of comprehensive reviews on the effect of modifying the adhesive, adherends, and joint configurations on the fatigue performance of bonded composite materials. Although in 1999, Goeij et al. [27] conducted a review on the impact of modification techniques on adhesive-bonded composite joints under cyclic fatigue loading, to the best knowledge of the authors, no further studies have explored the fatigue life of these joints from this perspective, while new techniques to enhance the fatigue life of bonded composite joints have been introduced in the literature. Hence, this review aims to provide a comprehensive overview of the existing literature on adhesive bonded composite joints under fatigue loading, focusing on three main aspects: 1. Adherend modification, 2. adhesive modification, and 3. joint configurations.

Furthermore, this review explores how modifying components and changing the joint configuration affects the fatigue and static loading behavior of adhesively bonded composite joints and attempts to compile and compare the resulting experimental data.

## 2. Crack Initiation Inspection

Fatigue damage is a common failure mechanism in adhesively bonded joints, which can significantly reduce the joint’s performance and lead to catastrophic failure if not addressed. Therefore, it is crucial to develop effective inspection and repair procedures to ensure the long-term reliability and safety of critical bonded joints. Detecting cracks and delamination in composite adherend structures can be challenging, as cracks in the adhesive tend to propagate into the composite adherends, leading to delamination. Delamination can spread quickly and cause catastrophic damage to both the adherend and the joint, making it more difficult to inspect composite-bonded joints compared to metallic joints. A comprehensive inspection approach should address failure at the interface, within the adhesive and delamination. However, implementing such an approach can pose significant challenges. As a result, precise monitoring is necessary to ensure the long-term reliability and safety of bonded composite structures. In line with this, researchers have investigated the fatigue behavior of adhesively bonded composite joints and proposed various methods for monitoring and detecting fatigue damage. These methods include non-destructive evaluation (NDE) using guided wave sensing, electrical resistance measurements [40], video microscopy, strain gauges (SGs), and the backface strain technique. However, the accuracy and effectiveness of these methods can vary depending on the type of joint and the location of the monitoring equipment. Further research is needed to develop more comprehensive and reliable inspection and repair procedures for critical bonded joints.

Palanisamy et al. [41] recommend using NDE techniques such as guided wave sensing to monitor composite lap joints. Such techniques enable not only the detection of damage but also the prediction of disbonded areas within the joints and the remaining useful life of the structure. Employing this technique, it becomes possible to detect and monitor the growth of cracks in adhesive-bonded joints, which can help identify potential safety risks before they result in catastrophic failures. They suggested that this technique can be used to develop a reliable and effective monitoring system for bonded structures (health monitoring), ensuring their safe and long-term use. Carboni et al. [42] conducted an experiment involving a single lap joint (SLJ) where both adherends were made of carbon fiber-reinforced polymer (CFRP). The joint was subjected to fatigue loading with a constant amplitude. Acoustic emission was used to continuously monitor the integrity and damage state of the joint during the test. Additionally, the test was periodically paused to conduct micro-computed tomography scans on the specimen. The findings indicate that by employing suitable data processing techniques and applying pattern recognition algorithms, acoustic emission monitoring proves to be an effective method for identifying and characterizing the progression of fatigue damage in adhesive-bonded joints [43].

**Figure 2:**
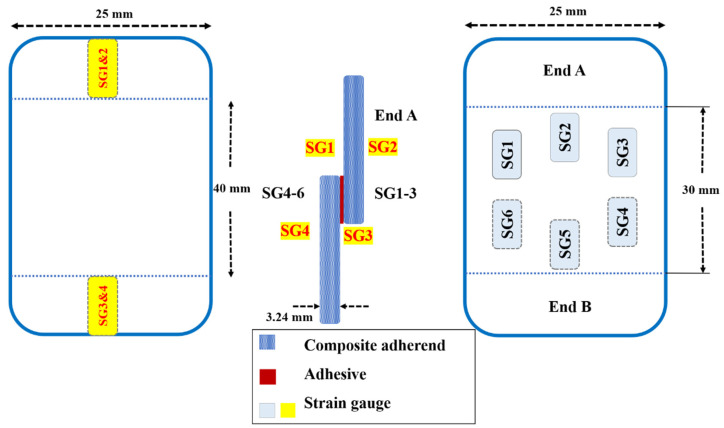
A schematic of SG placed (not to scale) (**A**) adapted from [49] outward overlap length and (**B**) adapted from [50] inward overlap length.

Quaresimin et al. [44] conducted fatigue tests on single-lap composite joints with different overlap lengths and monitored the damage using video microscopy and SGs. They defined crack initiation as the phase during which the adhesive failed and found that it could last between 20% and 70% of the fatigue life depending on the stress level and overlap length. Ishii et al. [45] and Zeng et al. [46] also used video microscopy to monitor fatigue damage in adhesive joints but used different definitions for crack initiation. According to Ishii [45], crack initiation was determined as the point at which the crack formed over the tip of the adherend corner present in the fillet. In contrast, Zeng et al. [46] identified the initiation point as the appearance of the first visible crack. Cheuk et al. [47] tested double-lap joints and identified two different crack initiation scenarios: Cracks initiated on the corner of the adherend and within the fillet using both video microscopy and SGs. Crocombe et al. [48] used the backface strain technique to detect crack initiation in SLJs for the first time. However, in subsequent work [49], it was revealed that the response of the backface strain was significantly influenced by its location. To enhance measurement accuracy, the authors suggested placing the strain gauges inside the overlap area. To optimize the position of the strain gauges, the relationship between variations in strain and increased fatigue damage should be examined. Therefore, the strain gauges should be positioned where the most significant changes in strain are observed due to varying crack sizes. This is essential to ensure the sensitivity of the backface strain gauge technique in detecting the initiation of fatigue cracks. Crocombe et al. [48] suggested employing the finite element method to obtain the strain variation as a function of crack growth length in adhesively bonded composite joints. They proposed utilizing this method to optimize the position of SGs. Nonetheless, it is important to note that this approach can exhibit variations based on different configurations and materials. For instance, as shown in Figure 2, unlike the findings of reference [49] suggesting that SGs should be positioned within the overlap region, Tang et al. [50] obtained satisfactory results by placing the SGs outside the overlap length.

While there is currently no research indicating the exact optimal quantity of SGs to utilize, it is significant to consider the failed SGs encountered during testing. To ensure an adequate and precise dataset, it is recommended to employ multiple SGs on each side rather than just one. Furthermore, in the case of adhesively bonded joints using a ductile adhesive, the backface strain gauge is not sensitive enough during the initiation phase due to plastic deformation.

## 3. Joint Configuration and Geometry

**Figure 3:**
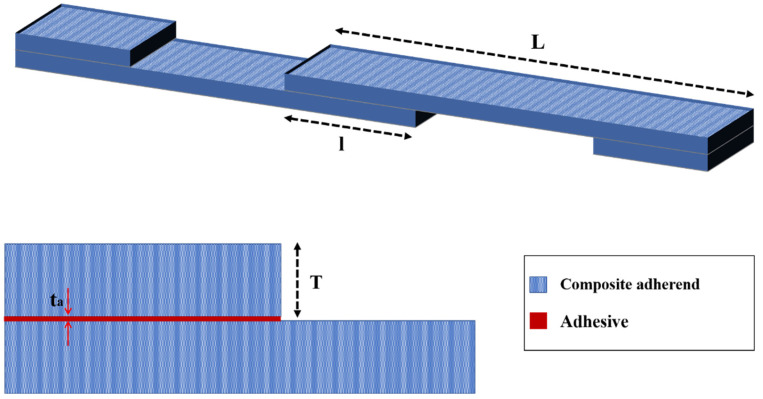
Design parameters of conventional SLJ.

**Figure 4:**
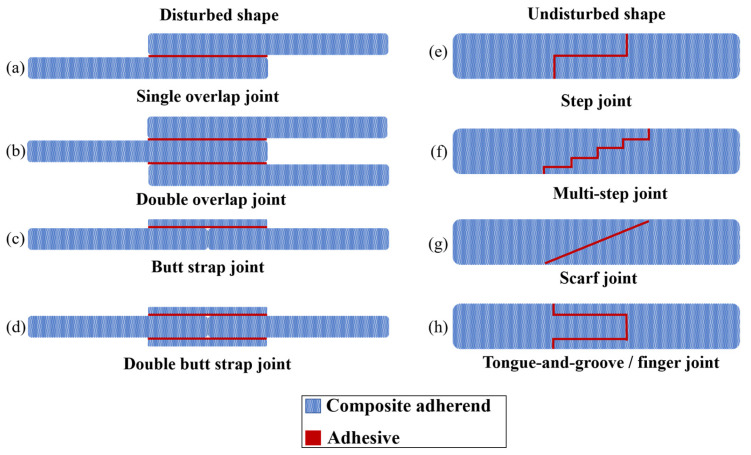
Different configurations of adhesively bonded joints. (**a**) Single overlap joint, (**b**) Double overlap joint, (**c**) Butt strap joint, (**d**) Double butt strap joint, (**e**) Step joint, (**f**) Multi step joint, (**g**) Scarf joint, (**h**) Tongue and groove/finger joint.

To enhance the fatigue strength of adhesively bonded composite joints, there are two potential approaches: Enhancing the properties of the adhesive or adherend materials, as suggested in references [51,52], and which are discussed in further detail in Section 4 and Section 5, or optimizing the geometry of the adhesive joints. This section aims to investigate the impact of joint configuration and geometry parameters, such as overlap length (*l*), overlap geometry (interface configuration), corner geometry, adhesive thickness (*ta*), and adherends thickness (*T*) on the fatigue behavior of composite adhesive joints (see Figure 3). By examining these key parameters, valuable insights can be obtained regarding how joint geometry can be strategically optimized to enhance the fatigue life of adhesive joints. This research is crucial for industries operating within the aerospace and automotive sectors, where the reliability and durability of adhesive bonds are critical for safety and performance. However, identifying the best adhesive joint configuration in terms of fatigue loading can be a very challenging task due to the existence of numerous configurations, as shown in Figure 4, and the absence of a comprehensive study that examines the behavior of each configuration under fatigue loading. 

**Figure 5:**
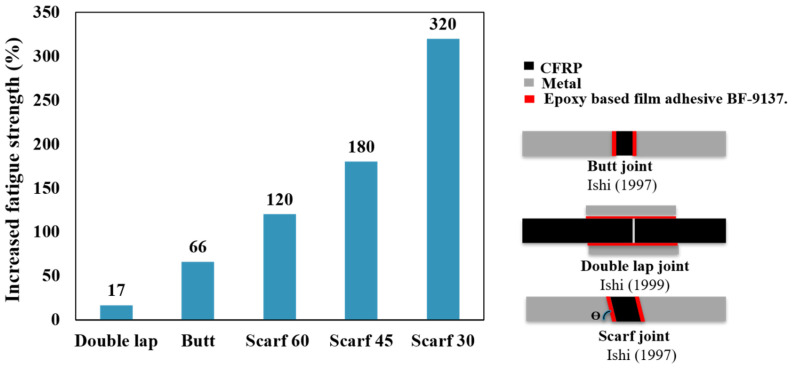
Fatigue strength (corresponding to 1 × 10^6^ cycle) improvement for different configurations in comparison to the SLJ (adapted from [35,45]).

The majority of studies evaluating the impact of the joint configuration on the fatigue behavior of adhesively bonded joints have focused on metal or dissimilar metal/composite materials as adherends. Only a few studies have specifically examined adhesive joints with composite materials as adherends. The fatigue strength of an adhesively bonded joint is affected by several factors, including the fatigue resistance of the materials involved. In the case of a joint made from dissimilar materials, such as CFRP and metal, the fatigue strength is primarily determined by the fatigue resistance of the CFRP plate or the adhesive layer. This means that the fatigue life of the joint depends mainly on the ability of the CFRP plate to resist cyclic loading without developing cracks or other types of damage. Ishii [35] developed a fatigue failure criterion for dissimilar adhesively bonded joints subjected to multiaxial stress. This criterion is used to estimate the fatigue strength of different configurations of CFRP-metal joints (single and double lap, butt, and scarf joints). The study findings indicated that the fatigue strength of double-lap joints was significantly superior to that of SLJs. Overall, the findings indicate that decreasing the scarf angle results in an improvement in the fatigue strength of scarf joints, driven by an increase in the bonded area. This is consistent with the conclusions drawn in other studies [53,54,55]. Scarf joints exhibited a higher fatigue life than butt joints of equal CFRP adherends thickness, regardless of the scarf angle. As the thickness of the CFRP adherends in the butt joint increases, the fatigue strength is reduced due to the volume effect of CFRP, and the dominant failure mode shifts from primarily delamination to cohesive failure. Figure 5 illustrates the increase in fatigue strength, specifically the load threshold at which the specimen has withstood over 10 million cycles, known as the endurance limit, in comparison to the SLJ.

To ensure comparability, the fatigue load is normalized by dividing it by the overlap area for each configuration. This adjustment takes into account the size of the adherends and the adhesive layer used.

There has been a limited amount of research conducted on the influence of different configurations and geometries of adhesively bonded composite joints on life under cyclic fatigue loads. Kim et al. [56] investigated the fatigue behavior of stepped lap joints in composite structures through static and fatigue tensile load tests. In all step lap joints presented in [56], fatigue cracks initiated at the end of the overlap and then propagated through the composite adherends, leading to the delamination of the substrates. As the length of the joint against the number of steps (steps ratio) increased beyond a ratio of 6, the fatigue life for each load level also increased. Conversely, when the steps ratio was reduced below 6, it significantly decreased the fatigue life. This means that a smaller step ratio had a negative impact on fatigue life. This indicates that the design characteristics of the joint, specifically the proportion of its overlap length to the number of steps, had a notable impact on its resistance to fatigue. Importantly, it was found that the fatigue endurance limit, which denotes the load or stress level a joint can endure for 1 × 10^6^ cycles without failing, corresponds to 30% of the static tensile load. The strength of the step lap joint relies on the shear stress present at the last step, as well as the stress that is distributed within the stepped lap joint, which is recognized to be controlled by the peak stress [57]. Once the end of the overlap section ruptures, the stepped lap joint can be considered an SLJ. As explained in more detail in the next section, with an increase in the length of the joint, the peel stress diminishes, correlating to the extended joint length. Furthermore, increasing the number of steps while keeping the bond length constant in step lap adhesively bonded joints can lead to an increase in fatigue strength due to several reasons, including the distributed load over a larger area, enhanced load transfer through the provision of an additional load path, the increase in effective bond length, and an interlocking effect. Collectively, these factors contribute to an increased fatigue strength in step lap adhesively bonded joints with an increased number of steps while maintaining a constant bond length. In general, stepped lap joints in composite structures can withstand high loads under both static and fatigue conditions. In designing a stepped lap joint, the first consideration should be the critical joint length, which is crucial for ensuring an effective bonded connection. The next consideration should be determining the appropriate number of steps needed to achieve the desired joint length.

### 3.1. Overlap Shape

**Figure 6:**
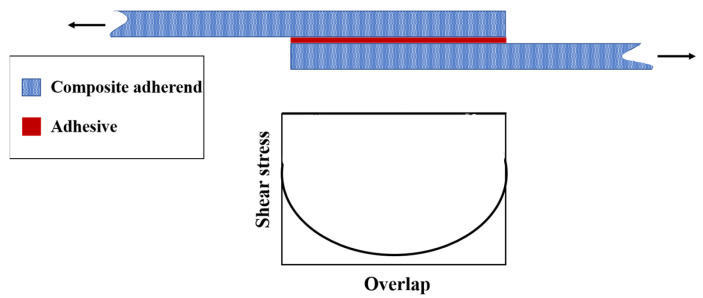
Shear stress distribution along the overlap length.

**Figure 7:**
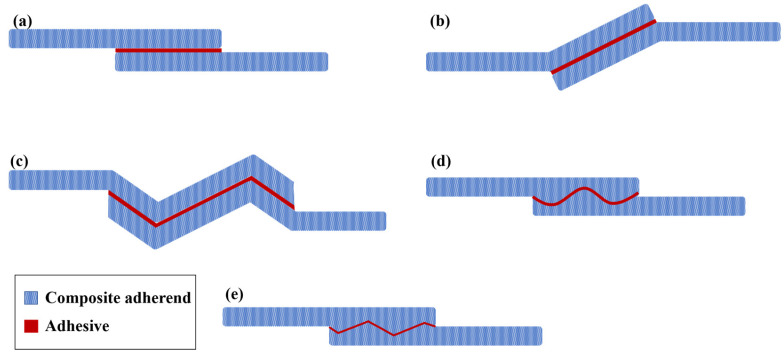
(**a**) Conventional SLJ, (**b**) Reverse-bent SLJ, (**c**,**e**) Wavy SLJ, and (**d**) SLJ with sinusoid interface (adapted from [70]).

In adhesively bonded SLJs, the free ends of the overlap region (overlap ends) experience stress concentrations (see Figure 6), which can lead to the failure of adhesively bonded joints under various loading conditions [58,59]. Several researchers attempted to create local compressive stresses at the ends of the adhesive layer by designing non-flat bonding surfaces [60,61]. To this aim, four primary geometric designs have emerged, including the reverse bent, wavy, sinusoid interface, and zigzag interface SLJs (Figure 7). Previous studies that examined these types of adhesive joints are summarized in the following paragraphs.

Boss et al. [58] observed that modifying the shape of the adherends (changing their thickness gradually) resulted in a decrease in peak shear and peel stresses in SLJs, leading to an overall improvement in the joint’s strength. Based on results consistent with Boss’ study [58], Pires et al. [62] and Maclaren et al. [63] concluded that changes in geometry should be at the overlap ends. This study aimed to enhance the uniformity of the stress distribution. To achieve this goal, the study proposed bent SLJs, in which the adherends were subjected to a bending moment opposite to that in conventional SLJs. As a result, the study demonstrated a significantly more uniform shear stress distribution. Despite the considerable advantages they offered, reverse bent joints (Figure 7b) with composite adherends have not received enough attention in the literature, due to the difficulty in precisely controlling the geometry of the adherends during the manufacturing process. The fatigue behavior of reverse-bent composite joints was investigated by Fessel et al. [64]. The study concluded that improvements achieved under static loading conditions can lead to even greater benefits in fatigue loading. By reducing peel stresses, the reverse bent joint experienced a significant increase in joint strength of up to approximately 190% in comparison with conventional SLJs, depending on the materials and fiber direction used. Additionally, improved fatigue performance was found for the reverse-bent joints, mainly due to reduced stress concentrations at the joint ends. The initiation of failure in the reverse-bent joint was not at singular stress points, resulting in a less complicated prediction of both static and fatigue strength. Overall, the combination of reduced peel stresses and decreased stress concentrations in the reverse-bent joint leads to improved joint strength and reliability. The efficiency of the reverse-bent joint configuration has been well studied and documented by multiple researchers [65,66,67].

A widely used method to enhance the mechanical interlocking of adherends involves creating a non-flat bonding surface on the adherends prior to the application of adhesive [68]. Ashrafi et al. [69] conducted a study in which they varied the shape of the joint interface while keeping the adhesive thickness constant. This was performed to ensure comparability and to determine the potential for improving the structural performance of composite SLJs. The usage of sinusoid interfaces (as depicted in Figure 7d) in glass fiber-reinforced polymer adherends revealed that the non-flatness of the interface had a notable influence on both the mechanical behavior and strength of the bonded joints. However, it was noted that modifying the angle of the wavy lap joint could result in higher strength and a more uniform distribution of stress [68].

**Figure 8:**
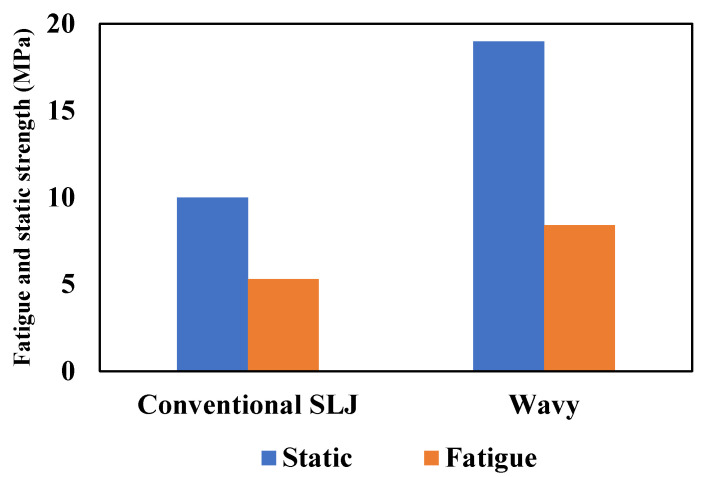
Effect of wavy interface on the fatigue and static strength of joints (adapted from [46]).

Zeng et al. [46,71,72,73] introduced an alternative form of adherend geometry called ‘wavy lap joints’ (see Figure 7c). In their studies, the fatigue performance of the wavy lap joint was compared to the conventional one, and comparative fatigue tests were conducted to demonstrate the advantages of the new wavy lap joint design. Firstly, static strength tests were performed on unidirectional composite adherends to compare the conventional flat joint and the wavy joint. Fatigue tests were then conducted at various load levels and frequencies. The test results showed that the wavy lap joint had a considerably longer fatigue life and higher strength than the conventional lap joint (see Figure 8). The analysis of the failure surface in a wavy joint under fatigue loading revealed that cohesive failure was the primary mode of failure. However, in the case of a conventional SLJ, unstable shear breakage was observed specifically in the middle section of the overlap length.

**Figure 9:**
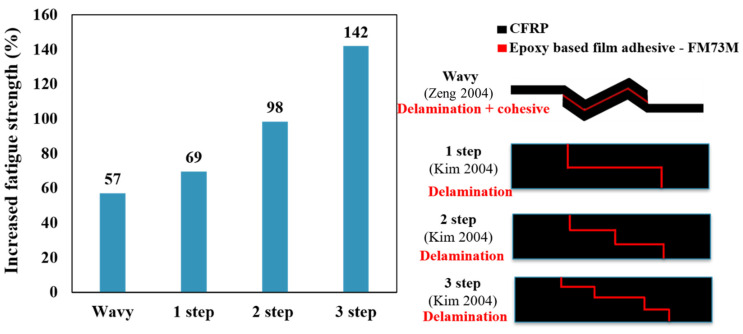
Fatigue strength improvement for different configurations compared to conventional SLJs (adapted from [46,56]).

The resulting improvement in fatigue strength for different configurations is depicted in Figure 9. To allow for direct comparisons in the fatigue performance, the fatigue strength is normalized by dividing it by the overlap area. This normalization takes into account the use of a similar adhesive for the various configurations presented. In conclusion, the wavy joint configuration can lead to improved joint strength compared to conventional SLJs, particularly under fatigue loading conditions. The reduction in peel stresses and stress concentrations at the joint ends are key factors contributing to these improvements.

The final aspect to consider is that utilizing non-flat overlap regions can enhance the fatigue strength and behavior of adhesively bonded joints, and the significance of locally modifying adherend geometry becomes even more crucial when joint configurations are limited by their application. However, it is essential to address the complexity of manufacturing and the lack of precise control over the geometry of composite adherends, as these factors can lead to variations and increased costs.

### 3.2. Overlap Length

Changing the overlap length between joint components can lead to changes in how stress is distributed within the joint. According to the available literature, it is crucial to consider a minimum overlap length to attain the best possible fatigue performance for the joints [27]. The impact of overlap length on the strength of composite joints is closely tied to the adhesive’s ductility. While it is widely recognized that a longer overlap increases joint strength when using a ductile adhesive, there is evidence that for a brittle adhesive, the failure load remains constant once a specific point is reached [74]. However, the rate at which this strength improves is influenced by various design factors like the materials used for the adherends and the adhesive [75]. Consequently, to achieve the maximum fatigue life for the same load level, the overlap length should be optimized for each configuration while considering the effect of other design parameters [76]. For instance, a model called the Genetic Algorithm Tensile Strength Estimation Model (GATSEM) was developed by Canyurt et al. [77] to estimate the fatigue and static strength of adhesively bonded finger composite joints as a type of step lap joint [78]. The model takes into account factors such as overlap length, bondline thickness, pre-stress near the free edges of the bondline, and the material types of the joining parts. The use of GATSEM enabled Canyurt et al. [77] to optimize the overlap length in order to achieve the highest possible fatigue life while considering the effect of other design parameters. It was found that the fatigue life of CFRP/CFRP configurations was 219% higher than the reference finger joint with fixed overlap length and bondline thickness. This indicated that GATSEM can be an effective tool for optimizing the design of adhesively bonded composite joints to enhance their fatigue life.

**Figure 10:**
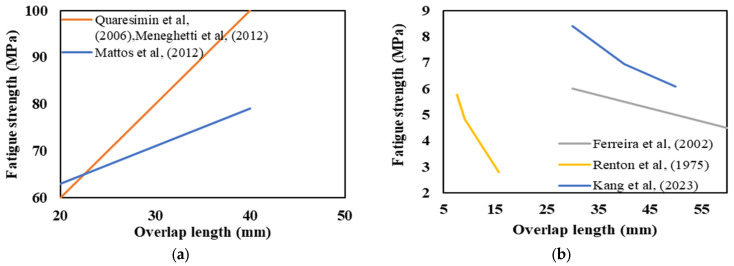
Opposite trend of the effect of overlap length on the fatigue strength of adhesively bonded composite joints. (**a**) Fatigue strength increased by overlap [44,83,84,85]. (**b**) Fatigue strength decreased by overlap [52,79,80].

The effect of overlap length on the fatigue behavior of adhesively bonded composite joints was first proposed in the early 1970s by Renton and Vinson [52]. Using a ductile adhesive, they found that the tensile ultimate load of single-lap composite joints increased with the length of overlap. The absolute value of fatigue load also increased with the increase in overlap length, as expected. However, the maximum normalized fatigue strength (fatigue load divided by bonded area) decreased as the overlap length increased. This means that the maximum load that can be sustained for a 1-million-cycle fatigue life is not directly and linearly proportional to the overlap length. Ferreira et al. [79] conducted fatigue tests on composite SLJs, and in line with some other studies [52,80,81], found that increasing the overlap length led to a reduction in normalized fatigue strength. In contrast, conflicting trends have been recorded in alternative research, as illustrated in [82,83,84,85], which have indicated that the fatigue strength of adhesively bonded joints improved when increasing overlap length (refer to Figure 10). 

**Figure 11:**
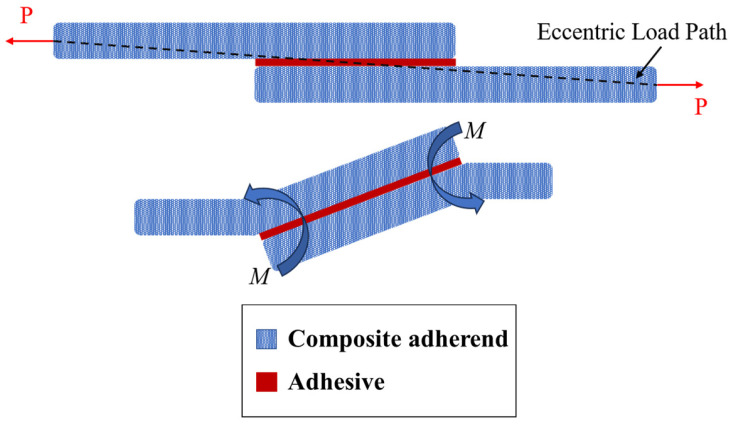
Free-body diagram illustrating the bending moment resulting from eccentric loading in a single-lap joint subjected to tensile loads.

SLJs experience slight bending due to the bending moment, generated by the applied tensile–tensile fatigue load (see Figure 11). Increasing the overlap length raises the bending distance, which can make failure more likely under fatigue loading [82]. Relying solely on classical theory to explain these conflicting trends, as seen in Figure 10, can be difficult. It is essential to also consider the failure initiation mode of the joints and the ductility of the adhesive. For example, in terms of adhesive brittleness, it is widely recognized that when using a brittle and rigid adhesive, increasing the overlap length results in higher stress concentration at the overlap ends. Consequently, this intensified stress concentration makes the occurrence of failure even more likely.

Single-lap joints that experience cohesive/adhesive failure under fatigue loading demonstrate an increased fatigue life with increasing overlap length, whereas joints that undergo adherend failure, such as delamination and fiber tear, exhibit an opposite or non-identifiable trend with respect to the overlap length. From another perspective, in cases where the overlap length exceeds the adhesive thickness by 10 times, it is more likely that the failure of the composite adherends would occur before any other failure modes [86].

### 3.3. Corner Geometry

**Figure 12:**
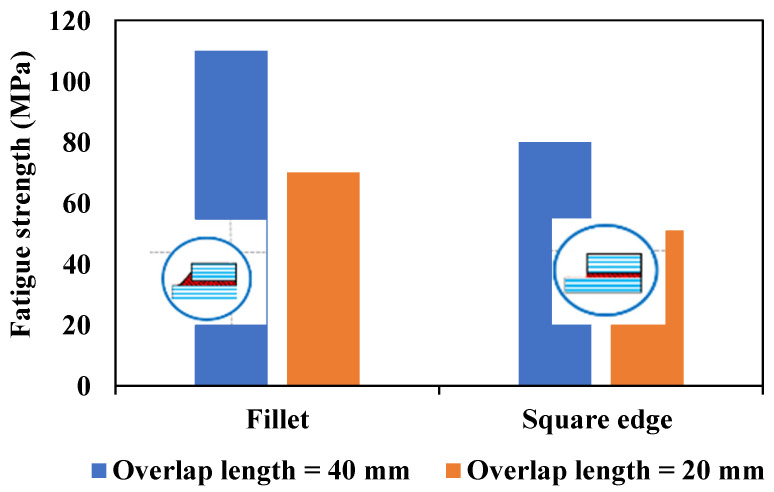
Effect of overlap length vs. corner fillet on fatigue strength [83].

**Figure 13:**
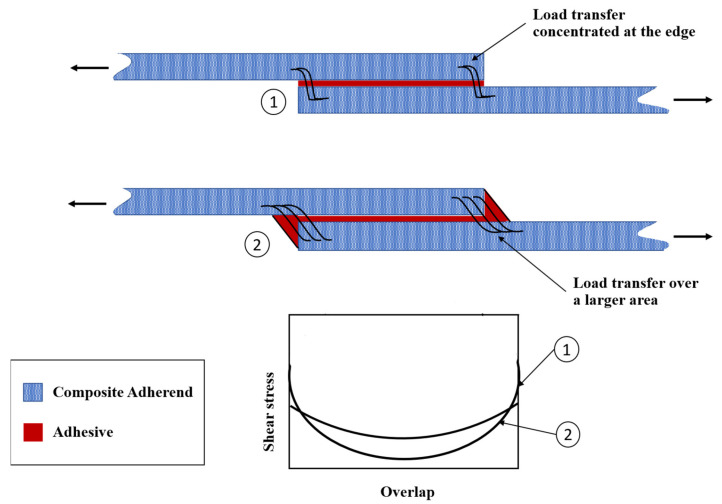
Effect of corner fillet on the load transfer path. ① Square corner ② Fillet corner (adapted from [89]).

Although the length of the overlap in adhesively bonded joints has a significant impact on both the initiation and propagation of fatigue crack growth, more so than factors like the corner fillet [83], as shown in Figure 12, the strength of adhesively bonded composite joints (the highest tensile stress that can be applied to the bonded joint without causing failure until 1 × 10^6^ cycles) has been enhanced by incorporating a spew fillet at the overlap ends. This can be explained by the reduction in the stress concentration within both the adhesive and the adherends [87,88]. Changing the joint-end geometry with a spew fillet spreads the load transfer over a larger area, resulting in a more uniform shear stress distribution (see Figure 13) [38]. In [83], the two-part 9323 B/A epoxy was used to prepare the tested SLJs. According to the results, for joints with fillets at bonding ends, failure was initiated within the adhesive fillet, or, in some cases, it was at the adhesive–adherend interface. Then interlaminar and intralaminar crack propagation was observed. On the other side, for the square corner joints, the initiation was shown to be interfacial at the adhesive/adherend interface while the crack propagation mechanism was similar to the joints with a fillet.

**Figure 14:**
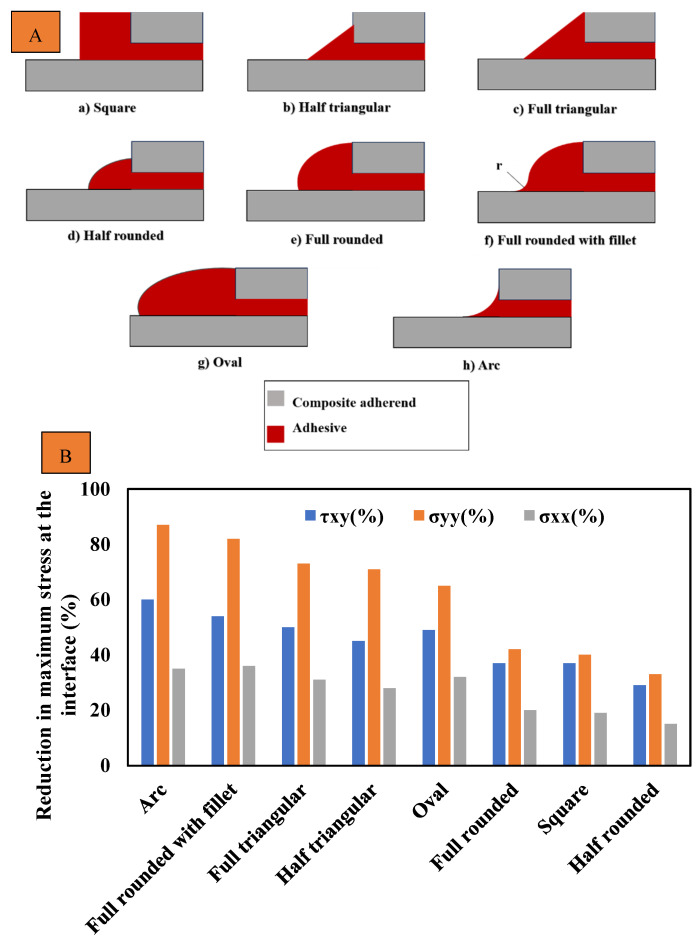
(**A**) Various fillet geometries, (**B**) decrease in maximum stresses at the interface between the conventional SLJ without a corner fillet and SLJs with the different spew geometries [87].

**Figure 15:**
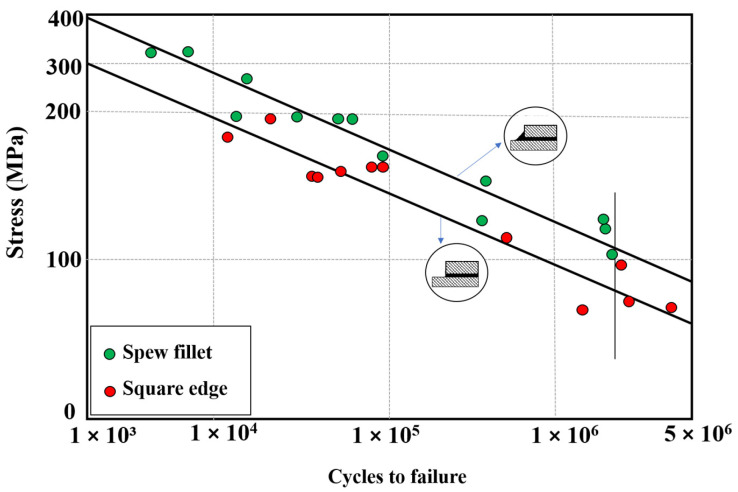
Effect of corner geometry (fillet) on the fatigue life and strength of SLJs (adapted from [44]).

The fatigue life and strength of joints are correlated with either peel stress, shear stress, or a combination of both, depending on the joint configurations [50]. Consequently, it would be reasonable that future studies on the fatigue life of adhesively bonded composite joints could benefit from referencing previous research on optimizing the corner geometry of joints to achieve higher static strength. Lang and Mallick [87] conducted a study that involved eight different corner geometries for glass-epoxy composite SLJs, including full and half triangular, full and half rounded, and full rounded with fillet, oval, and arc. Their findings indicated that shaping the spew to provide a smoother transition in joint geometry could significantly diminish stress concentrations. As shown in Figure 14b, the full-rounded configuration with fillet and arc spew fillets resulted in the highest percentage reduction in maximum stresses (nominal in the direction of the applied load (x), as well as perpendicular (y) and shear), while half-rounded fillets showed the least reduction. Furthermore, increasing the size of the spew was also found to reduce the peak stress concentrations (see Figure 14). Quaresimin and Ricotta [44] conducted a series of static and fatigue tests to evaluate the effect of different geometry parameters on the strength of composite SLJs. As evident from Figure 12 and Figure 15, the use of fillets increases the average fatigue strength by approximately 27–55%. This increase in fatigue strength due to the use of fillets can be explained by the lower stress concentrations corresponding to the spew fillet corner geometry compared to a square edge [90]. 

### 3.4. Adhesive Thickness

The thickness of the bondline (also known as the adhesive thickness) is an important parameter that can affect the strength of bonded joints. Several experimental studies have shown that for both metallic and composite SLJs, increasing the bondline thickness (for a value higher than approximately 0.2 mm) can lead to a reduction in joint strength due to higher peeling stress [28,91]. This is also due to the fact that a thicker bondline can lead to a decreased ability of the adhesive to effectively transfer stresses between the adherends, potentially resulting in premature joint failure. In contrast, classical analytical stress-based solutions of SLJs predict that increasing the bondline thickness would result in an increase in the strength of the joint [50,92]. According to Niranjan [93], increasing the bondline thickness raises the likelihood of the presence of imperfections, such as voids and microcracks, within the joint. These imperfections can also cause the joints to fail prematurely.

**Figure 16:**
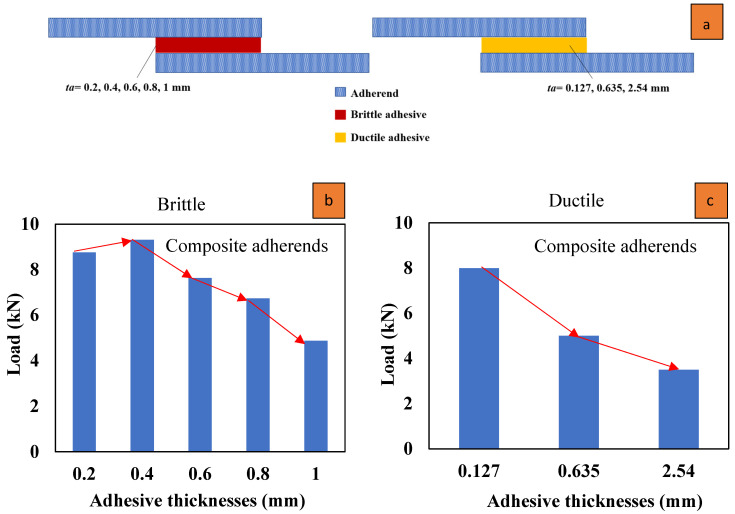
(**a**) Configuration of adhesively bonded composite joint with brittle and ductile adhesives and different adhesive thicknesses. (**b**,**c**) Composite SLJs strength as a function of adhesive thickness for brittle and ductile adhesives, respectively (adapted from [95,96]).

Thus, it can be concluded that there exists an optimal value for adhesive thickness. The impact of bondline thickness on the static strength of SLJs varies depending on the adhesive’s ductility [94]. Taib et al. [95] investigated the effect of adhesive thickness on the strength of adhesively bonded composite SLJs. It was indicated for ductile adhesives (Hysol EA 9359.3) that as the bondline becomes thicker, the strength decreases (see Figure 16). A similar trend was shown for metallic adherends [94]. For brittle adhesives (such as LP-1Q-EX, epoxy), as Diharjo et al. [96] reported and as is shown in Figure 16, increasing the bondline thickness results in an increase in static strength, which continues until it reaches a certain threshold [96]. Beyond this point, the strength starts to decline (as reported in other studies [86,97]). Consequently, the response to the bondline thickness variations is primarily driven by the adhesive’s ductility and is not significantly influenced by the adherends’ material.

**Figure 17:**
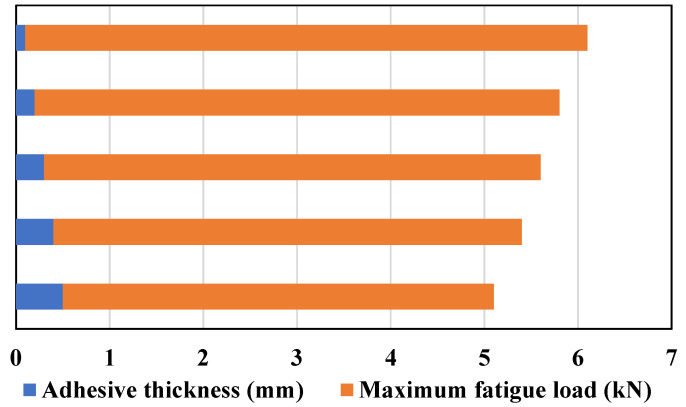
How the adhesive thickness affects the highest load that can be applied to the bonded joint without causing failure in 1 × 10^6^ cycles (adapted from [81]).

Only a few studies have attempted to investigate the impact of bondline thickness on the fatigue behavior of bonded composite joints, and among them, only concrete evidence of a decrease in fatigue strength with an increase in bondline thickness was reported [50,98]. Renton et al. [99] experimentally studied the parameters that drive an efficient design. They found that the choice of adhesive should be influenced by the expected loading history of the joint. Under static loading, the adhesive should have high ultimate strength values in both tension and shear while, for fatigue applications, the fracture toughness of the adhesive should also be taken into account [99]. Some studies have indicated that when it comes to structural epoxy adhesives, fracture toughness and fatigue resistance are considerably influenced by the thickness of the adhesive [100]. Increasing the bondline thickness leads to a reduction In the rate of fatigue crack growth, as well as an increase in its fracture toughness across the typical range of thickness employed in bonded joints (i.e., less than 0.25 mm) [86]. The effect of adhesive thickness depends largely on whether it is smaller or larger than the optimal thickness specific to the material, and a unique trend cannot be observed for all cases [27]. In Figure 17, it is demonstrated that as the adhesive layer becomes thicker, the anticipated lifespan of the joint diminishes. This prediction, which is performed for composite adherends joints, is supported by the experimental results provided by Crocombe [101], in which an increase in adhesive thickness was found to be associated with a decrease in the strength of the joints, where aluminum adherends used. It is recognized by the classic theory that when the thickness of the adhesive increases, the peel stress also increases, leading to a decrease in the fatigue life [92]. While the adhesive thickness is increased, the length of the “moment arm” over which the applied force acts is effectively extended and makes a higher moment arm, subsequently leading to increased peel stresses at the edges of the adhesive bond. To support this, considering that peel stress is influenced by multiple factors, it becomes necessary to conduct a numerical investigation into the impact of adhesive thickness on peel stress. In [50,97], finite element analysis revealed that the peel stress increased with greater adhesive thickness.

**Figure 18:**
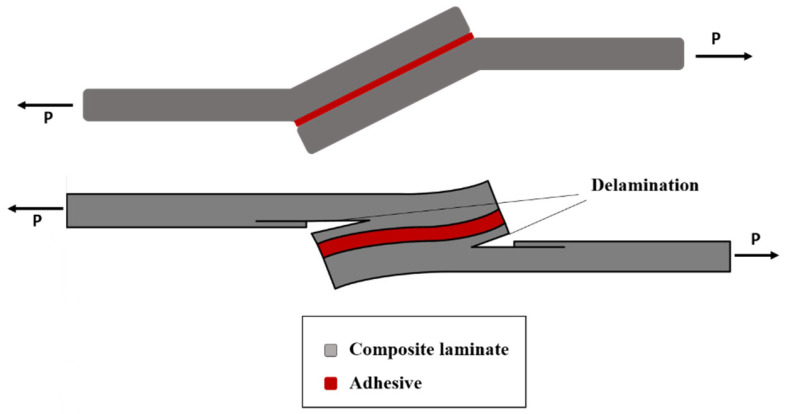
The correlation between the thickness of the adhesive and the level of peel stress, and its consequent effect on delamination.

The thickness of the adhesive also affects the failure mode so that failure occurs within the adhesive layer, and with an increase in thickness, the failure mode changes and delamination is raised due to an increase in peel stresses [97]. As previously discussed, it has been observed that peel stress rises in response to an increase in adhesive thickness. Given that peel stress significantly impacts the composite adherends at the ends of the overlap (where peel stress is most pronounced), this leads to a heightened risk of deamination, as depicted in Figure 18.

## 4. Adherend Modification

This section examines the impact of various modification methods applied to composite adherends on the fatigue behavior of adhesively bonded composite joints. Since this review primarily focuses on composite materials, Section 4.1 is specifically dedicated to analyzing the fatigue behavior of composite materials. After providing a concise overview of this topic, the investigation shifts towards an in-depth examination of composite adherends, which have been extensively studied in the context of fatigue performance.

### 4.1. Composite Materials under Fatigue Loading

Fatigue life improvement techniques for composite materials have been widely discussed since they were first introduced in the 1950s [102,103,104]. Several parameters, such as control of the fiber type and size, stacking sequence, fiber volume fraction, manufacturing methods, and the use of hybrid composites, are known to influence the fatigue life of composite materials and thus have been extensively investigated [105,106,107]. 

**Figure 19:**
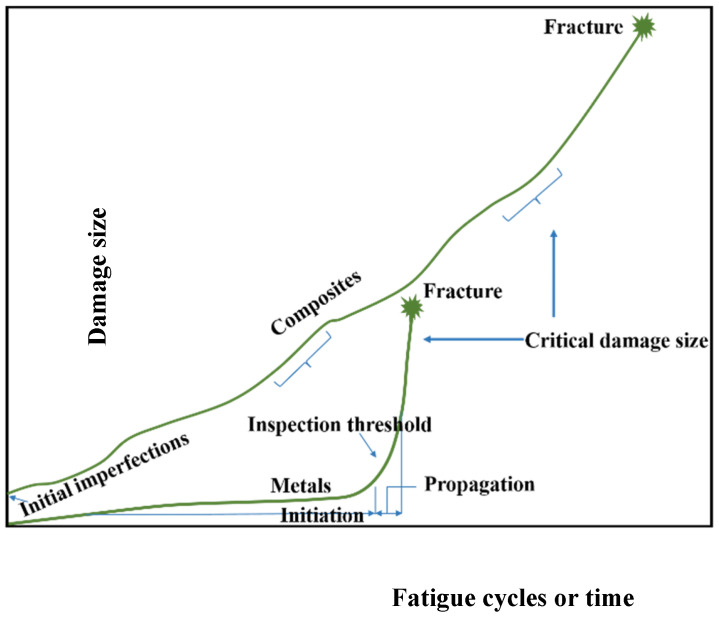
Difference between the fatigue failure pattern of composites and metals (adapted from [111,112]).

Crack initiation and propagation in composites differ from metals. The paths taken by cracks are more intricate and diverse, and the presence of cracks alone does not represent the entire structural damage. It has been observed that composite materials experience multiple forms of damage, such as delamination, matrix cracking, fiber failure, and void growth. Any of these damage modes or any combination of them may be experienced in a particular structure, and it is challenging to predict in advance which mode will prevail and ultimately lead to failure [108]. Hence, even though it is possible to detect and characterize all the damages that occur within a composite material, accurately quantifying their impact on fatigue life has proven to be exceptionally challenging [108]. Figure 19 illustrates the contrasting damage evolution between composite materials and metal structures. In metals, fatigue failure is typically characterized by the initiation of cracks, which subsequently propagate in a predictable manner until failure, while there has been different fatigue failure in composite materials including delamination, matrix cracking, debonding, fiber failure, and void growth [109]. There was an initial expectation that composite materials would exhibit greater damage tolerance compared to metals, depending on the orientation of the laminate and the specific loading conditions. The primary fatigue damage mechanisms observed in cross-ply laminates under tensile–tensile cyclic loading are transverse cracking, delamination, and fiber breakage. Additionally, in the 0° ply block, longitudinal cracking may also occur alongside fiber breakage [110].

**Figure 20:**
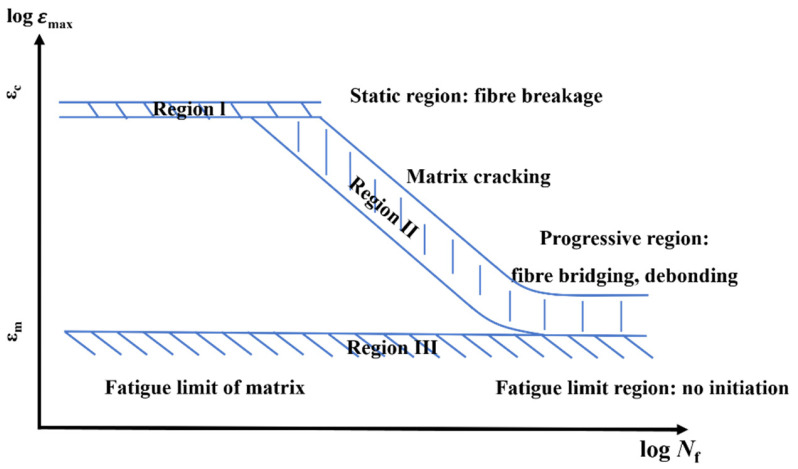
Relationship between strain and fatigue life for unidirectional composites. Region I: Initial phase of fiber failure. Region II: Interfacial shear breakage and matrix cracking. Region III: Fatigue limit (adapted from [111]).

Analyzing the impact of individual components on the fatigue performance of composite materials can yield valuable insights. Since the fiber and matrix experience different stresses under cyclic loading, while the strain remains the same for both, Talreja et al. [113] introduced a novel concept involving the examination of the strain behavior of each component as a function of fatigue life (ε-N curve) (as depicted in Figure 20). Fibers play a crucial role as the primary load-bearing components in composite materials, typically occupying a volume fraction ranging from 30% to 70%. Variations in the fiber volume fraction and properties, especially stiffness, can lead to changes in the stress–strain relationship of the composite material. In the case of commonly used carbon fibers, commercially available options exhibit different levels of stiffness and strain failure. These variations in fiber properties have a notable impact on the shape of the ε-N curve, which represents the relationship between strain and the number of fatigue cycles, and consequently affects the overall fatigue life of the composite material [114]. Region I in Figure 20 represents the initial phase of fiber failure, characterized by non-progressive failures that remain within a specific scatter range during the first half-cycle of load. Region II is related to interfacial shear breakage and matrix cracking, which can occur simultaneously. Fiber type selection significantly impacts region II of the ε-N curve. In particular, when subjected to the same fiber strain, high-modulus (HM) fibers endure higher stress levels compared to low-modulus (LM) fibers. Consequently, composites containing HM fibers are expected to experience lower levels of fatigue degradation. It is important to note that fatigue failure in composites cannot occur until cracks initiate in the matrix. Therefore, incorporating stiffer fibers into the composite structure makes sense as they can effectively impede crack propagation, thereby delaying fatigue failure.

**Figure 21:**
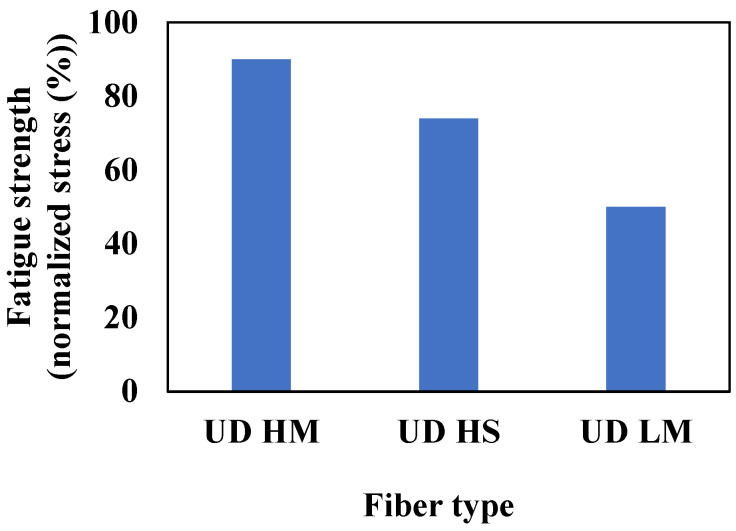
Normalized fatigue endurance: Investigating the strength for infinite composites life (1 × 10^6^ cycle). LM: Low modulus, HM: High modulus, HS: High strength (adapted from [115]).

The influence of fiber type (High modulus—HM, tensile modulus: 350–600 GPa; tensile strength: 2.5–5 GPa [114], low modulus–LM, tensile modulus: 40–200 GPa; tensile strength: 1–3.5 GPa [114]) on the fatigue behavior of UD composite laminates has been investigated in [115,116]. As shown in Figure 21, in line with the previous explanation, it is observed that high-modulus (HM) fibers tend to display greater normalized fatigue strength in unidirectional (UD) composites. The fatigue strength is normalized by static strength for each condition. Regarding the damage modes, it is observed that high-modulus (HM) fibers are more susceptible to sudden and catastrophic failures. On the other hand, low-modulus (LM) fibers tend to experience more progressive damage over time [117]. The correlation between fiber stiffness and failure mode can be explained by examining the stored energy within the material. Catastrophic failure occurs as a consequence of the sudden release of a significant amount of stored energy once the matrix fails. Conversely, progressive failure emerges due to stress localization, leading to the gradual and incremental release of localized pockets of stored energy.

The influence of fiber size on the fatigue strength of composite materials is widely recognized. Longer fibers might exhibit different mechanical properties, such as lower flexibility or reduced resistance to stress concentrations, which can impact their fatigue performance [114,118]. Furthermore, the interaction between the fibers and the surrounding matrix material may vary depending on the fiber length, resulting in variations in load transfer and mechanisms for initiating and propagating damage. Sabiston et al. [119] evaluated the impact of fiber length distribution (FLD) on the fatigue properties of CFRP. Their findings were consistent with the results reported by Karger-Kocsis and Friedrich [120] who demonstrated that increased fiber length leads to an extended fatigue life. Conversely, Meneghetti et al. [121] reported a different trend for the relationship between fiber length distribution and fatigue behavior that can be attributed to several factors, one of which is the specific lengths of the fibers being considered. Sabiston et al. [119] also reported that using 10 mm fibers resulted in a decreased fatigue life under fully reversed loading, compared to using 1 mm fibers. However, Meneghetti et al. [121] may have utilized different fiber lengths in their investigation.

**Figure 22:**
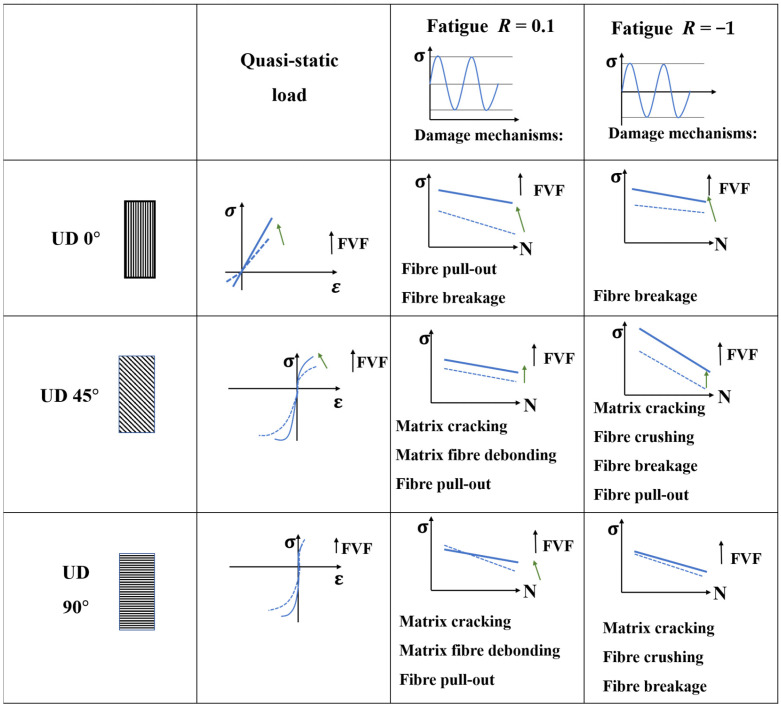
Summary of the conclusions of Brunbauer’s study [123] on the effect of fiber volume fraction on the static, fatigue, and failure mechanisms of different fiber-oriented composites.

The fatigue performance of composites can also be influenced by two other significant factors: Fiber volume fraction (FVF) and fiber orientation [122]. Pinter and Brunbauer [123] investigated the effect of various FVFs (30% and 50%) on the fatigue failure mechanism of epoxy CFRP. Static and cyclic fatigue loading tests were conducted on unidirectional composites that were manufactured with fiber orientations of 0°, 45°, and 90°. According to Figure 22, which is a summary of their results, in both tension and compression, the static strength is higher for samples with higher FVF. In unidirectional (UD) specimens with a 90° fiber orientation, the fatigue tests (both tension and compression) were primarily affected by the fiber volume content, particularly in the high cycle fatigue regime. This is due to the shift in damage mechanisms from fiber pull-out at higher stress levels to matrix cracking at lower stress levels [124,125]. 

**Figure 23:**
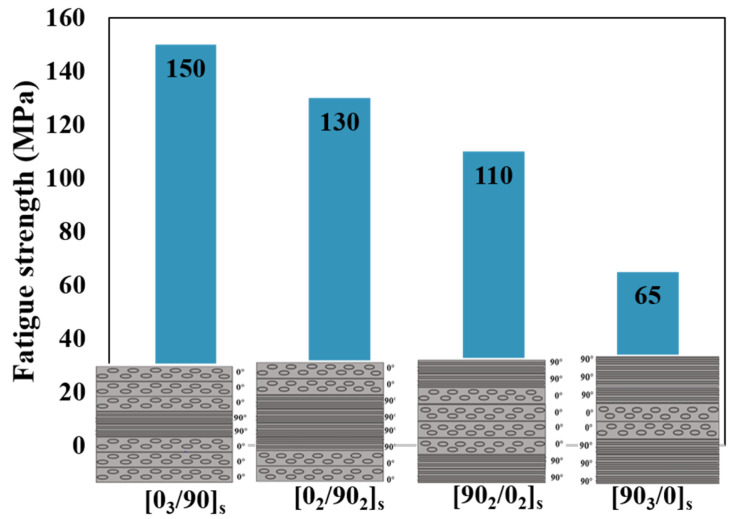
Effect of stacking sequence and 0° layer distance from the middle plane on fatigue strength (adapted from [126]).

Roundi et al. [126] conducted an experimental analysis to examine how different stacking sequences ([0_2_/90_2_]_s_; [90_2_/0_2_]_s_; [0_3_/90]_s_; [90_3_/0]_s_) affect the fatigue properties of glass/epoxy composites. Fatigue strength and life were evaluated through cyclic tensile tests. Higher fatigue strength and longer fatigue life were exhibited in the case of the [0_3_/90]_s_ stacking sequence, as illustrated in Figure 23. Mandell and Samborsky [127], in a vast experimental study, investigated different stacking sequences including balance angle plies ([0]_3_, [±45]_3_, [±50]_3_, [±60]_3_, [±80]_3_, [±90]_3_) and [90/0/±45/0]_s_, [±45/0_2_/45]_s_, [0/±45/0]_s_, [90/±45/90]_s_, [0/±45/0_2_/45]_s_. A comparison of the results obtained led to the conclusion that the unidirectional laminate has the highest fatigue strength. Sharma et al. [128] examined the impact of rearranging the position of (0°/90°) and (±45°) plies within an eight-layer symmetric and asymmetric woven glass fiber-reinforced composite laminate. The symmetric laminate had a stacking sequence of A [(0°/90°)/(±45°)_2_/(0°/90°)]_s_, while the asymmetric laminate had a stacking sequence of B [(0°/90°)/(±45°)_2_/(0°/90°)//(±45°)/(0°/90°)_2_/(±45°)]. The effects were investigated through fatigue testing subjected to stress levels ranging between 90% and 50% of their ultimate flexural strength. A longer fatigue life was experienced when the (0°/90°) ply was on the outer sides of the symmetric laminate. In general, the fatigue strength of composite materials can be enhanced by increasing the percentage of the 0° layer in the composite lay-up and by increasing the distance of the 0° layer from the middle plane (see Figure 23). The reason for this is that the 0° layer is responsible for carrying the majority of the load in the direction of loading. By increasing the percentage of the 0° layer, more fibers are aligned with the loading direction, which increases the strength of the composite material under such loads. Additionally, by raising the distance of the 0° layer from the middle plane, it becomes possible to have a greater moment arm of the 0° layer, which then increases its ability to resist bending stresses. This configuration effectively distributes the load across a greater volume of the composite material, reducing stress concentrations and enhancing fatigue resistance [129,130,131].

### 4.2. Hybrid Composite Laminate

**Figure 24:**
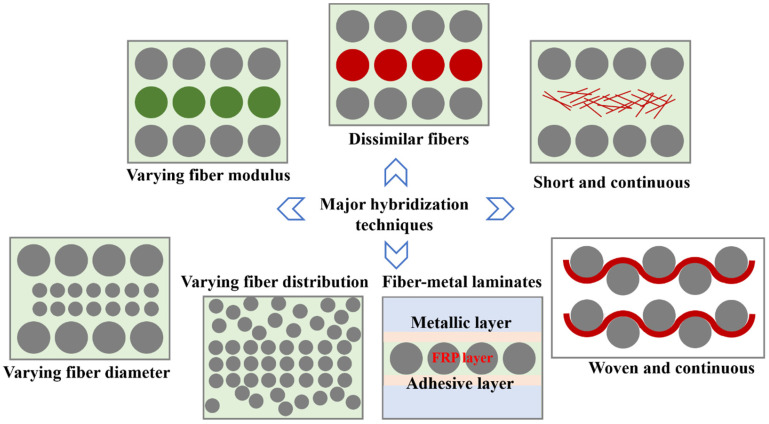
Schematic representation of various composite laminate hybridization techniques (categorizing hybrid fiber types and arrangements). Different colors used shows the difference in fiber materials (adapted from [136,144]).

The fatigue damage mechanisms of non-hybrid composites have been extensively studied and documented in the past [132,133,134]. In the case of hybrid composites, which involve the use of two or more fiber types, the failure mechanism undergoes several changes [135,136]. Hybrid or modified composites can be formed by combining fibers of varying modulus and/or diameter, either by using similar fibers or by incorporating dissimilar fibers into the composite structure [136] (see Figure 24). In the past, various types of hybrid composites have been developed by combining different kinds of fibers. These include mixtures like glass/carbon [137], Kevlar/carbon [138], Kevlar/glass [139], and synthetic/natural fibers [140]. These combinations were aimed at enhancing material properties and cost-effectiveness. The research presented in [141] demonstrated that a hybrid material combining a glass fiber-reinforced polymer (GFRP) and CFRP exhibited a lower S-N slope when compared to pure GFRP and pure CFRP across various fatigue load levels. The impact of hybridization on the fatigue life of composite materials depends on various factors, such as the loading condition (R ratio), fiber orientation [142], and fiber volume fraction [143]. In some cases, hybrid composites may exhibit improved fatigue life and strength compared to non-hybrid composites, depending on the specific combination of parameters mentioned.

The thickness of individual layers is a crucial aspect when designing hybrid laminates. In recent decades, researchers have investigated the phenomenon known as the size effect, which relates to the influence of ply thickness. Numerous studies have focused on experimentally evaluating the impact of the thickness of lamina under quasi-static loading conditions [145,146,147]. However, only a limited number of researchers have specifically explored the use of ultra-thin plies in their investigations. The utilization of thin plies within a cross-ply stacking sequence has been extensively studied in [148,149] focusing on the investigation of their impact on the fatigue behavior of composite materials. They concluded that the utilization of thin-ply can decrease the slope of the S-N curve for the laminate and, evidently, enhance the fatigue life [149]. París et al. [150], to clarify the concept of size effect, offered a physical explanation of failure mechanisms observed during quasi-static tensile loading. By adopting an energetic approach, they investigated the progressive development of damage mechanisms within the weakest lamina, specifically the 90° ply block. Their findings indicated that reducing the thickness of the transverse damage within this lamina can be advantageous. In other words, thinner 90° ply blocks exhibited less detrimental transverse damage. This beneficial feature of thinner 90° ply blocks under fatigue loading has received limited attention in existing studies. Therefore, further research is required to comprehensively analyze and understand the implications and advantages of reducing the thickness of the 90° ply block in the context of fatigue loading (effect of scale factor). 

Generally, in terms of non-hybrid composite laminates, those produced with the highest-performing material typically exhibit the most favorable quasi-static and fatigue behavior. The CFRP laminates have notably high specific mechanical properties, along with impressive fatigue life, surpassing that of GFRP laminates. However, even CFRP composites frequently undergo catastrophic failures due to brittleness, offering limited to no opportunity for repair and maintenance. This challenge can be addressed through the use of thin-ply hybrid composites, where fibers of similar material but varying diameters or moduli are employed [151]. Hybrid composites provide a relatively higher ductility. This approach leads to a more stable delamination growth. However, carbon fibers are still somewhat brittle and come at a significant cost. Furthermore, hybrid materials were developed with the aim of cutting down the expenses associated with using costly reinforcements in composites. This was achieved by employing a portion of more affordable, lower-quality fibers into the hybrid laminate, while still maintaining the main advantage and properties of the original composite. Moreover, hybrid materials were created with the intention of enhancing the characteristics of a composite without causing a significant impact on the overall cost. The process of hybridization is anticipated to yield novel materials that blend the properties of their individual components due to standard mixture principles. Nevertheless, hybrid composites exhibit superior properties compared to those predicted by the rule of mixtures. This phenomenon is referred to as the ‘hybrid effect’. However, as mentioned before, in some cases, hybridization shows a reduction in fatigue life and strength. 

### 4.3. Stacking Sequence and Interface Ply Orientation of Adherends

It is widely accepted that modified composite laminates exhibit superior quasi-static and fatigue behavior compared to conventional composites alone, as concluded in the previous section. Despite this acknowledgment, the impact of modifying the composite adherends on the fatigue life of adhesively bonded composite joints has not received the same level of attention as that of the composites themselves. Existing research has primarily focused on examining how various factors such as the orientation of the adhesive/adherends interface layer, adherends stacking sequence, adherends stiffness and thickness, and the applied surface treatment affect the fatigue performance of adhesively bonded composite joints under static loading, using stiffer adherends, which is achieved by increasing the number of 0° layers or placing them closer to the outer surfaces and enhances joint strength [152,153].

**Figure 25:**
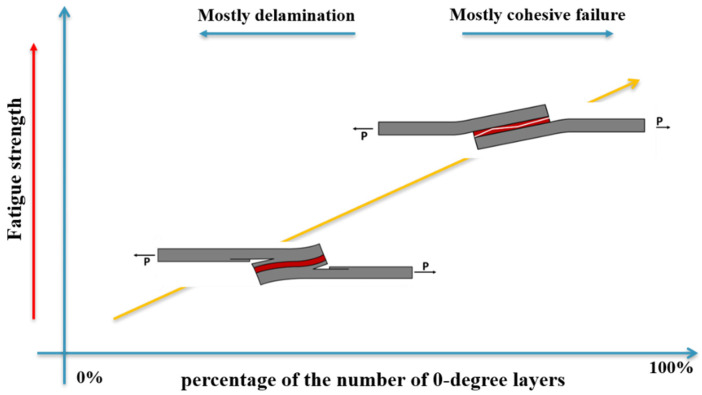
Effect of proportion of 0-degree layer through the thickness of adherends on fatigue strength [52,79,83,154].

Renton and Vinson [52,99] were among the first to investigate this issue, examining adherends made of glass/epoxy laminated with unidirectional, [0°], and multidirectional [45/0/−45/0] specimens. Their findings indicated that fatigue strength deteriorates as the nature of the adherend changes from unidirectional to multidirectional. This means that, with the same number of cycles, the angle ply pattern can sustain only 60–90% of the load carried by the full 0° adherend. In the first case ([0°]), cohesive failure occurred within the adhesive, while the failure mechanism and crack path were observed to occur within the adherend when a 45° layer was used as an adhesive adjacent layer. This can be attributed to the elevated interlaminar and normal stresses within the resin of the 45° ply [154]. In the study conducted by Ferreira et al. [79], a 30% increase in fatigue strength was reported due to a change in the stacking sequence from [+45/−45/+45/0]_s_ to [0/0/0/0]_s_. To summarize, the fatigue strength of a laminate is generally enhanced when stiffer adherends are used (see Figure 25). Purimpat [155] described an experimental technique involving the stacking of quasi-isotropic quasi-homogeneous (QIQH) sequences, which can effectively separate the local (plies orientation adjacent to the adhesive) and global (stiffness modification) properties of laminates. The main goal of this approach was to modify the orientation of plies near the adhesive bonding without changing the global elastic behavior of the adherends. Since the static strength was evaluated in this study, from this point of view, investigating the adhesively bonded composite joint under fatigue loading is crucial. Meneghetti et al. [83] analyzed the fatigue failure mechanism with a specific focus on the adhesive/adherend interface layer orientation (0°, 45°). In more detail, the effect of the adhesive adjacent layer fiber orientation on the fatigue life of joints in two initiation and propagation crack growth stages was examined. They reported that the fatigue crack propagation life was extended when using an interface layer consisting of a 45° orientation compared to using an interface layer with a 0° orientation due to the more complex crack growth pattern in the presence of 45°. However, this has a negligible effect on the fatigue life. The damage propagation pattern significantly changes by the ply orientation of the adhesive/adherends interface layer. When the interface joint is oriented at 0°, cracks propagate along the interface between the adhesive and adherend. However, if there is a 45° interface present, the evolution of crack propagation becomes significantly more complex, so that after nucleation, cracks may propagate at the adhesive/adherend interface or become interlaminar delamination. Figure 25 displays SLJs manufactured using composite adherends with various stacking sequences, as opposed to SLJs where all adherends are oriented at 0°. As evident from the results, the proportion of 0° layers has a direct impact on the increase in fatigue strength and influences the mode of failure [52,79,83,154,156]. It can be explained by increasing the stiffness of joints with more 0° layers. It is important to highlight that the impact of the stacking sequence in Figure 25 is assessed under the assumption of maintaining the other parameters constant. For instance, in the study referenced as [157], where both the stacking sequence and surface roughness were examined simultaneously, it was observed that the [[±45]_4s_]_s_ stacking sequence exhibited greater fatigue strength compared to configurations with all 0° adherends. This improvement in fatigue strength was attributed to alterations in the surface roughness of the adherends.

Johnson and Mall [152,153], along with Rodriguez [154], conducted studies involving lap strap joints using composites, with a specific focus on the impact of the interface layer orientation on fatigue behavior. In their research, Johnson and Mall explored different stacking sequences like [0/±45/90]_s_, [±45/0/90]_2s_, and [90/±45/0]_2s_ for both the strap and lap configurations. On the other hand, Casas-Rodriguez examined the stacking sequence of [(0/−45/+45/0)_2_]_s_. The total strain energy release rate was employed to compare fatigue failure in different configurations of Johnson and Mall’s [152,153] study, instead of a stress-based threshold, due to the varying number of adjacent 0° layers adjacent to the adhesive in the different configurations. These researchers observed that joints with 0° or ±45° plies at the interface exhibited nearly identical values for the strain energy release rate and fatigue strength [158]. Keeping the stiffness of adherends (through the stacking sequence) and the adherends thickness constant were found to be the primary factors affecting fatigue strength. With cyclic adhesive separation remaining the main contributor to fatigue damage initiation, it is anticipated that joints with various ply orientations at the interface would show comparable fatigue strength. Li et al. [75] examined the influence of adherend thickness on single-lap, double-lap, and scarf composite joints. They found that increasing adherend thickness resulted in a greater failure load for all these configurations, indicating higher strength for single- and double-lap joints, but conversely, lower strength for scarf joints was observed. In [81], it has been demonstrated that with an increase in adherend thickness, there is an expected corresponding increase in the number of cycles required for joint failure. However, it is worth noting that there are still no available experimental data to validate this prediction. This underscores the clear need for additional research in this specific area.

**Figure 26:**
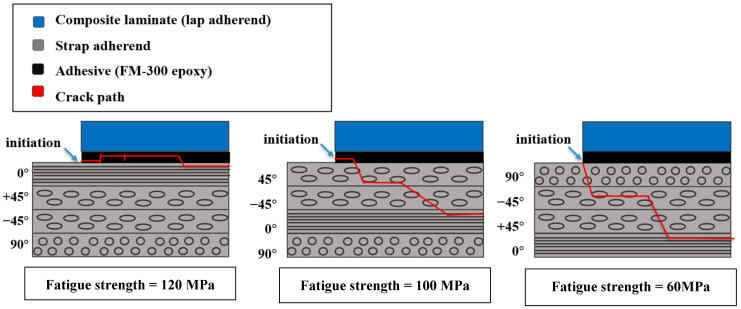
Changing crack path due to adhesive adjacent composite adherends layer orientation (0/+45/−45/90), (45/−45/0/90), (90/−45/+45/0) (adapted from [158,159,160]).

As shown in Figure 26, as long as the interface plies are placed at 0°, debonding occurs within the adhesive region in a cohesive manner and final failure occurs in the 0° adjacent layer. However, in the case of 45° interface plies, debonding happens through a combination of cyclic debonding in the adhesive and delamination in the ±45^°^ plies of the laminate adherend. In more detail, the crack continues to grow until it reaches the layer with 0° orientation and then the final failure occurs. For specimens with 90^°^ plies, damage initiates with transverse cracking in the 90^°^ layers of the strap adherend, and then progresses through delamination failure until it reaches the first 0^°^ ply in the strap adherend (see Figure 26).

In general, based on existing literature, it is obvious that the stacking sequence and the orientation of the adhesive adjacent layer have an impact on the fatigue crack path. Consequently, this affects both the fatigue strength and fatigue life. Nonetheless, the impact of these factors on fatigue strength varies significantly based on the joint configuration. As previously discussed, using unidirectional fibers aligned with the load direction results in greater fatigue strength. The least favorable stacking sequence involves a perpendicular orientation, particularly evident in SLJs. However, in lap strap joints, these effects tend to diminish.

### 4.4. Surface Treatment

In order to achieve optimal adhesion in adhesively bonded composite joints, it is imperative to conduct a comprehensive surface treatment that adequately eliminates all contaminants from the bonding surfaces while simultaneously increasing the surface area available for bonding. The strength of the joints significantly relies on the effectiveness of the surface treatment in ensuring strong adhesion at the interfaces.

The success of the bonding process can be ensured by enhancing the wettability of the surfaces, which involves increasing their surface energy and chemically activating them to improve adhesion. Surface energy is a measure of the energy required to increase the surface area of a material. Increasing the surface energy of a material signifies that the molecules on the surface possess elevated potential energy. This heightened energy state makes the surface more chemically receptive and enhances its level of adhesion. Chemical activation is closely related to surface energy, as the goal of chemical activation is to increase the surface energy of a material. Chemical activation can be achieved by various methods such as plasma treatment [161], corona discharge [162], ion irradiation [163], laser activation [164], solvent cleaning [165], and flame treatment [166,167], which improve the wettability of the surface by increasing the surface energy of the material [168].

Alternatively, surface roughening can be used to increase the mechanical interlocking between the adhesive and the adherend, further enhancing the strength of the bond [169,170]. Roughening the surface can be achieved by various methods, such as sand and bristle blasting [31], grit blasting [171,172], and the peel ply technique [173,174]. This process creates a rough surface topography, which increases the surface area and provides more points of contact for mechanical interlocking between the adhesive and the adherend. The resulting mechanical interlocking increases the strength of the bond, making it more resistant to shear and peel stresses [175].

The effect of surface treatment on the static and fatigue strength of composite materials has gained wide attention, but studies on the fatigue life of adhesively bonded composite joints are less prevalent [15,176]. Wingfield [165], one of the earliest researchers in this field, emphasized the importance of surface treatment in the adhesive bonding of composites. He explained and identified several fundamental mechanisms that impact the effectiveness of surface treatment, which included (i) removing the particles at the surface boundary layer by removing contaminants and oxidized layers, (ii) enhancing the wetting ability of low-energy surfaces, (iii) modifying the surface chemically, and (iv) increasing the surface roughness to enhance mechanical interlocking and expand the bondable surface area. He suggested that the surface treatment should be carried out on a thin layer of the surface while ensuring that the properties of the bulk composite are not affected.

The surfaces of treated adherends are relatively fragile. Studies have revealed that even contact with clean work gloves can diminish the surface energy [177]. As mentioned before, surface roughening can increase mechanical interlock and result in greater adhesion at the adherend/adhesive interface. However, excessive roughness can adversely affect the fatigue strength of the composite adherends [178]. The relatively higher fatigue endurance might be obtained due to favorable surface roughness made by sanding treatment [178]. Thäsler et al. [179] investigated the static strength and fatigue life of single-lap composite joints by applying two different surface treatments, including plasma jet and vacuum blasting, and also generating surface roughness by combining release foil/peel at a constant maximum force level of 4 kN for all configurations. In the case of a specific type of release foil, vacuum blasting proved to be the most effective, resulting in a fatigue lifetime of 72,000 cycles. Plasma treatment yielded a fatigue lifetime of 16,000 cycles, while the untreated joints had a fatigue lifetime of only 43 cycles.

**Figure 27:**
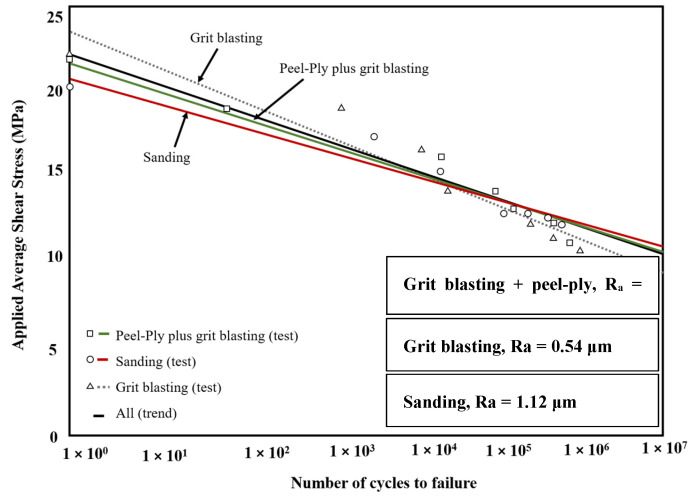
SLJ fatigue test results (sanding, grit blasting (GB), peel-ply plus grit blasting (PPGB)) [174,181].

Liu et al. [180] investigated the fatigue performance of SLJs composed of CFRP and aluminum adherends bonded with a bi-component epoxy adhesive by applying a grit blasting treatment with 180 mesh sandpaper. It was observed that the cohesive strength was stronger than the interface strength between the aluminum adherend and the adhesive during fatigue loading. However, in the case of quasi-static loading, cohesive failure occurred. Additionally, the study revealed notable differences in crack initiation and propagation between quasi-static loading and fatigue loading conditions. Similarly, in a study by Park et al. [174], the fatigue life of single-lap composite joints was investigated by comparing the surface energy and roughness of composite adherends prepared using grit blasting, hand sanding, and peel-ply plus grit blasting. They concluded that the peel-ply plus grit blasting treatment resulted in the highest surface roughness and energy, as measured by 3D image analysis and a contact profilometer. Although the static strength of all surface treatments was relatively similar at approximately 22 MPa, the sanding treatment exhibited a lower strength value of 20.5 MPa. The fatigue strength was found to be 55.4% of the static strength for the sanding treatment after 1 × 10^6^ cycles, and 47.5% and 50.3% for grit blasting and peel-ply plus grit blasting, respectively (see Figure 27). The scale of roughness can have a significant impact on fatigue resistance. This may either decrease or increase the resistance to fatigue. As mentioned before, the roughness should be optimized to gain higher fatigue strength. For instance, Park reported [174] that a surface treatment using peel-ply plus grit blasting, which achieved a higher roughness value (10.31 μm), did not show higher fatigue strength compared to hand sanding with a roughness of just 2.45 μm. A surface with an optimized amount of roughness can result in the highest fatigue life due to several reasons, including an increase in the bonded area, enhancement of mechanical locking, and retardation of crack growth. Conversely, a surface that is too rough can lead to the formation of voids and stress concentration at the adhesive/adherend interface [181]. Surface treatments applied to adherends can impact the behavior of composite bonded joints during fatigue and static loading. However, this influence could depend on factors like the joint configuration and surface treatment parameters (such as surface roughness). As a result, these treatments might either enhance or degrade the strength of the joints.

## 5. Adhesive Modification

In composite joints, the adhesive layer is often the limiting factor in joint performance due to its weaker properties compared to the adherend in most cases [182]. An analysis of the stress distribution in an adhesively bonded joint provides insight into how the adhesive properties impact the behavior of the joint. The strength of a joint is directly related to the stresses that the adhesive layer experiences [183,184,185]. Adding fibers or particles into the adhesive or using a bi-adhesive approach within the adhesive layer of the joints are recognized methods for enhancing the fatigue strength of adhesively bonded composite joints [186]. This section of the review paper focuses on exploring these techniques in further detail.

### 5.1. Nano-Reinforced Adhesive Layers

To enhance the performance of adhesively bonded joints, one potential approach involves incorporating nanoparticles into the adhesive, even in small amounts (1–1.5%) [23]. The strength of a joint reinforced with nanomaterials is influenced by various factors that affect the adhesive’s behavior and its ability to transmit stress to the composite adherends. One of the key factors is the type of adhesive, which can be rigid, flexible, or toughened. Another significant factor is the ratio and type of the added nanostructure, including materials such as carbon nanotubes and graphene. Furthermore, the characteristics of the nanostructures such as their size, shape, and aspect ratio, can impact the dispersion and alignment of these structures within the adhesive [187]. Apart from these aspects, other factors such as the loading conditions, preparation of the bonding surface, and the curing process can also play a role in the strength of the joint reinforced with nanomaterials. Hence, considering all these variables is crucial in the design of nano-reinforced adhesive joints to achieve optimal performance.

**Figure 28:**
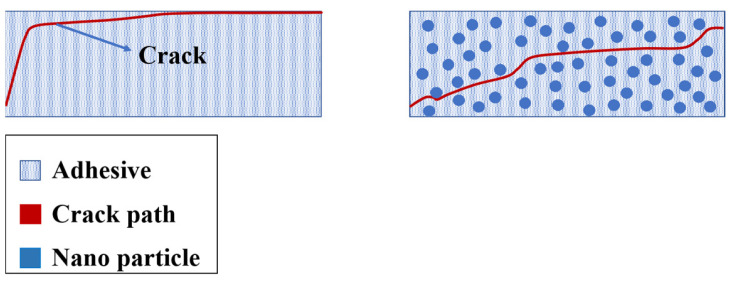
Crack path affected by nanoparticle (greater resistance to crack initiation and propagation due to more complex crack path).

**Figure 29:**
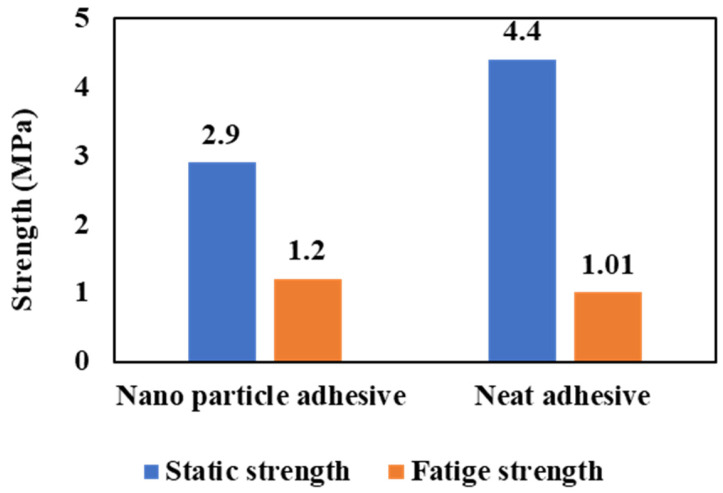
Effect of adding nanoparticles to the adhesive layer on the static and fatigue strength of SLJs (adapted from [190]).

Using nanofillers to modify adhesives has been shown in many studies to enhance the mechanical properties such as strength, stiffness, toughness, and fracture energy of adhesive joints, making them suitable for bonding composites [187,188] by improving stress transfer between the adhesive and the composite adherends [23]. This improvement can be attributed to the enhanced wettability of the adherends by the adhesive [189] and also the greater resistance to crack initiation and propagation due to the more complex crack path (see Figure 28), achieved through the utilization of nanoparticles. Moreover, due to their size, they can penetrate the voids within the composite adherends, increasing the mechanical interlock and consequently enhancing the joints’ strength. These changes in behavior change the failure mode from adhesive failure at the interface to cohesive failure within the adhesive, ultimately leading to a more effective transfer of load to the adherends. In contrast, some studies have indicated that the addition of nanofillers can lead to a decrease [190] in the strength of the joints. This decrease may be attributed to an increase in the brittleness of the adhesive and its glass transition temperature (*T_g_*) [23]. For example, Kang et al. [190] indicated that in the case of a composite/aluminum adhesive joint without carbon nanotubes under tensile loading, there was an occurrence of interlaminar failure within the composite adherend. This type of failure occurred because the adhesive was strong enough to transfer the load effectively between the adherends. Conversely, adding carbon nanotubes to the adhesive resulted in adhesive failure at the interface between the adhesive and the composite adherend, as observed. This can be attributed to the degradation of interfacial bonding between the carbon nanotubes and the adhesive, which reduced the strength of the adhesive and its capacity to transfer stress to the composite adherend. Furthermore, the analysis of fracture surfaces did not reveal any significant difference in the behavior of the joints under fatigue compared to static loading conditions. The effect of adding nanoparticles to the adhesive layer on the static and fatigue strength of SLJs is shown in Figure 29. The addition of 2 wt% carbon nanoparticles resulted in a minor enhancement of around 18% of the tensile–tensile fatigue strength, but with a decrease of approximately 51% in static strength (see Figure 29) [190]. The conclusion drawn is that the impact of incorporating nanoparticles on the static and fatigue strength of adhesively bonded composite joints is not necessarily uniform; it varies depending on multiple factors including the type of adhesive and nanoparticles as well as the effect of nanoparticles on the ductility of the adhesive [191,192,193]. 

**Figure 30:**
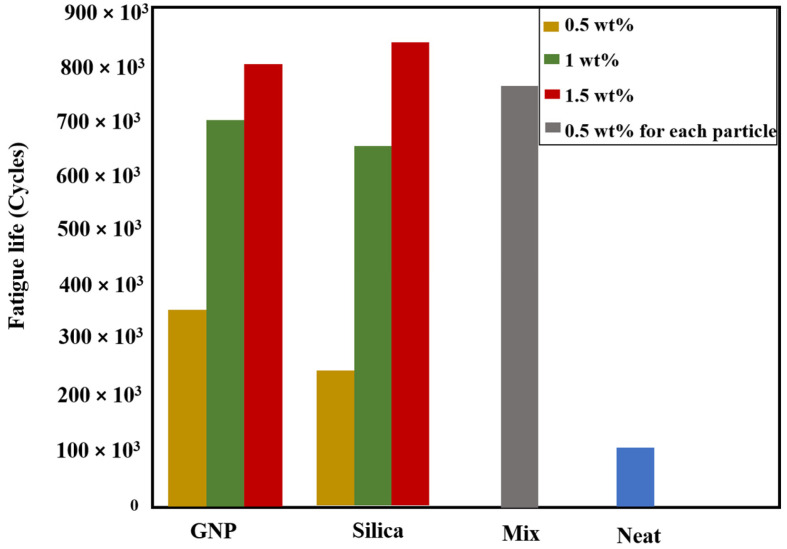
Comparative fatigue life analysis: Assessing the influence of nano-particle content, specifically examining fatigue performance at approximately 30% of static failure load (adapted from [194]).

Zamani et al. [194] evaluated the effect of adding nanoparticles (graphene and silica) to Araldite 2015 adhesive on the four-point bending fatigue behavior of aluminum-composite SLJs. The study involved the preparation of four different adhesive groups, namely (I) the neat adhesive, (II) the adhesive reinforced with nano silica particles, (III) the adhesive reinforced with nanographene particles, and (IV) a combination of both types of nanoparticles (with a content of 0.5 wt% for each particle). The reinforcement content for groups II and III varied from 0.5 wt% to 1.0 wt% and 1.5 wt%, respectively. Results revealed that the adhesion of the adhesive with the nanoparticle is higher than that of the neat adhesive. Specimens containing nano-particles experienced cohesive failure while the neat epoxy specimens exhibited an adhesive failure mode. Furthermore, the use of a combination of graphene nanoplatelet (GNP) and nano silica particles with a weight percentage of 0.5% each in the adhesive resulted in a higher static failure load and longer fatigue life compared to using each type of reinforcement individually at 0.5 wt% and 1 wt% (see Figure 30). Using the nanoparticle mixture with a lower weight percentage of each particle is a more cost-efficient option than using them separately with a higher weight percentage. Consequently, assessing the weight percentage of each particle should be considered precisely in order to be able to use the benefit of nanoparticles. Bali and Topkaya [195] investigated the performance of SLJs that were reinforced with adhesives containing GNPs under tensile and tensile–tensile fatigue loading conditions. The study indicated that the addition of GNPs can enhance the strength of the adhesive. However, the highest static strength was achieved in samples containing 1% GNP as the optimum value [23]. The highest fatigue strength was observed in samples containing 0.50% GNP, indicating that there is an optimal weight percentage of GNPs that can improve the fatigue performance of the adhesive. In [196], adhesives with and without glass bubbles (microspheres) were evaluated for SLJs with composite adherends, and the specimens were subjected to various tension–tension fatigue loadings with a maximum load level of 90% of the bond shear strength. It was found that the addition of glass bubbles in the adhesive significantly improved the tensile strength and fatigue life of the SLJs. However, Khashaba et al. [197] found that while the incorporation of multi-walled carbon nanotubes MWCNTs and SiC in the scarf adhesive joints (SAJs) resulted in an improvement in their fatigue life, the fatigue life of the SAJs reinforced with Al_2_O_3_ decreased. This work was followed by another [36], where adhesive joints were fabricated using an epoxy adhesive system that was modified with 1.5 wt% SiC nanoparticles. This adhesive was used to join two woven carbon fiber composite adherends at a scarf angle of 5°. Adding SiC nanoparticles into the adhesive layer showed an improvement in the tensile strength and fatigue limit of SAJs by 26.1% and 22%, respectively, compared to unmodified joints. 

Considering all of the explanations presented above, the process of reinforcing materials with nanoparticles is seen as a process that involves several variables that influence the properties of the resulting composite, including the dispersion of nanoparticles, their structural features, and their length and diameter. Although numerous studies [198] have suggested that adding nanoparticles to adhesives can increase fatigue strength and lifespan, the type and content of nanoparticles used should be carefully considered to achieve optimal results [197].

### 5.2. Mixed Adhesive Layers

**Figure 31:**
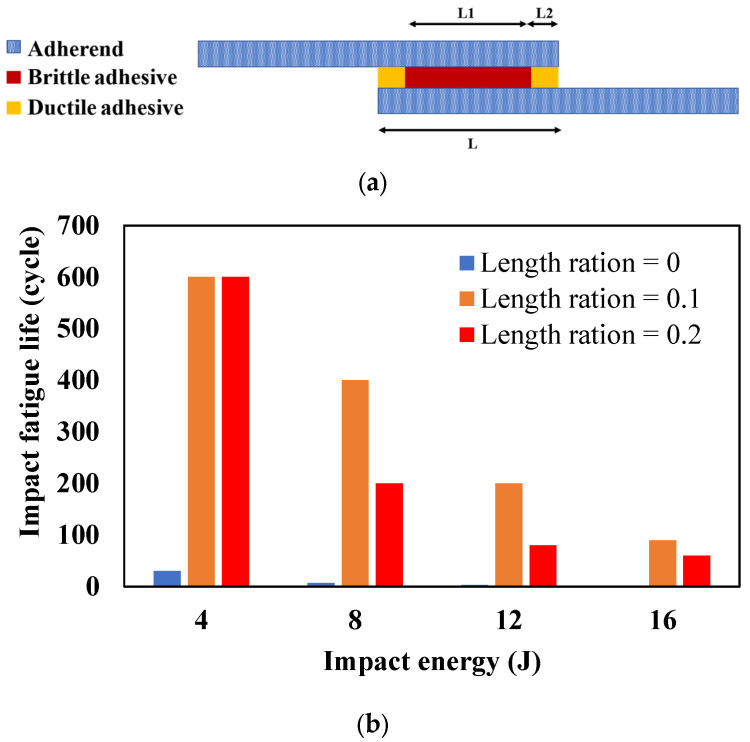
(**a**) Schematic of mixed adhesive joints. (**b**) Impact fatigue life assessment of mixed adhesive SLJ under varying impact energies: Exploring the effects of different impact levels on fatigue life of mixed adhesive joints [21,203].

Previous studies have proposed the use of mixed modulus adhesive joints, which consist of two different types of adhesives (one ductile and one brittle), to improve the stress distribution and fatigue strength of adhesively bonded joints [199]. In this approach, the stiff and brittle adhesive is placed at the center of the overlap, while a low-modulus adhesive is applied at the edges where stress concentrations are more likely to occur. Figure 31a shows the schematic of mixed adhesive joints and the combination of two different adhesives [200]. Utilizing a mixed adhesive is a viable option for applications that experience harsh environmental conditions and involve dissimilar adherends with notable variations in properties [201]. Flexible adhesives are preferred for their resistance to peeling, fatigue, crack propagation, and impact loads [202]. However, they typically have lower cohesive strength. Brittle adhesives, on the other hand, have a higher modulus and lower toughness. In [203], the bi-adhesive technique to enhance the performance of SLJs made of composite and metal materials under impact fatigue loading conditions was proposed. An experimental analysis was conducted to evaluate the impact fatigue life of composite-metal joints using a mixed-adhesives layer. The findings demonstrated that the impact fatigue life of bonded joints could be significantly enhanced simply by employing two different adhesives in the overlap region, following the mixed adhesive layer technique. Furthermore, it was observed that the optimal length ratio of the adhesives, i.e., the fraction of the overlap size occupied by the ductile adhesive, depended on the joint’s stiffness (adherend material) and was more pronounced for bonded joints with lower stiffness. Figure 31b illustrates how varying impact levels affect the fatigue life of mixed adhesive joints, taking into account the length ratio of the mixed adhesive used. Akhavan-Safar et al. [204] investigated the mixed adhesive joints under cyclic low-energy impacts and reported the impact energy vs. impact fatigue life. Similarly to the other study [201], it was observed that the impact fatigue life of bi-adhesive joints is highly influenced by the length ratio. The results demonstrated that the impact fatigue life of the joints improved significantly by employing the mixed adhesive technique. The experimental results indicated that even small changes in the length ratio of the two adhesives in the joint had a significant effect on its endurance to impact fatigue.

As far as the authors are aware, no previous studies have specifically focused on investigating the cyclic fatigue behavior of mixed adhesive joints. This research gap presents an interesting opportunity to explore the effects of employing this method in adhesive bonding. The study may involve comparing the fatigue performance of mixed adhesive joints with those of conventional adhesive joints and other bonding methods. This comparative examination offers valuable insights into the relative benefits and limitations of mixed adhesive joints, while it also highlights their potential utility in diverse industries that encounter high-cycle fatigue scenarios.

### 5.3. Other Modification Methods

**Figure 32:**
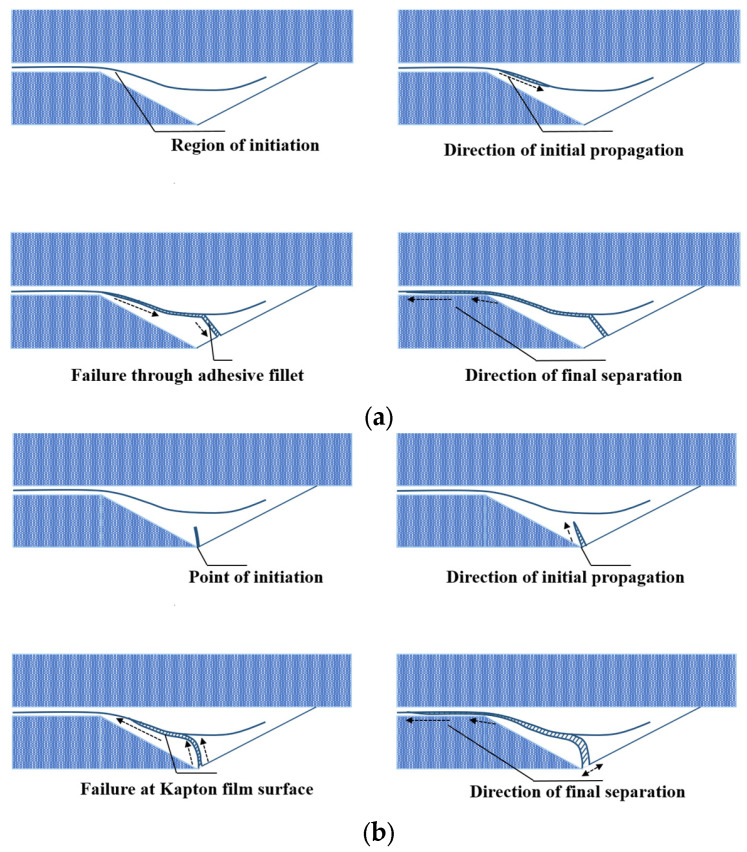
Fatigue crack growth in modified sample employing (**a**) Kapton film cloth. (**b**) Nylon cloth, the arrows show the damage propagation path (adapted from [205]).

Potter et al. [205] carried out a comprehensive experimental and numerical study of a novel method to modify the performance of adhesively composite bonded joints under quasi-static and fatigue loads. Their idea was to alter the design of adhesive joints in composite structures so that any potential failure occurs within the bond interface and does not propagate into the adjacent composite laminates. To achieve this goal, nylon mesh and Kapton film were embedded within the adhesive. The modified joints were tested under tension–tension fatigue. The fatigue strength of the samples was higher compared to that of the unmodified samples as illustrated in this context. In more detail, the modified joints recorded a fatigue life of 10 million cycles at approximately 40 percent of their static strength, whereas the unmodified samples only endured 20 percent of their static strength within the same 10 million cycles. As shown in Figure 32a, the failure mechanism observed in the Kapton-modified specimens was initiated at the tip of one of the adherends similar to that of the unmodified joints. Subsequently, the crack propagated towards the adhesive fillet, extending in the direction of the Kapton layer. Once the crack reached the Kapton layer, it was deflected and followed a path along the Kapton layer. Failure in nylon-modified specimens was observed to initiate at the interface between the nylon and adhesive, as shown in Figure 32b. The separation occurred along the nylon/adhesive interface, initially extending toward the adhesive fillet. With further extension, the crack was deflected into the adhesive and eventually led to complete separation of the adhesive fillet from the inner adherend. This failure sequence was consistent with the predictions from Finite Element Analysis (FEA), as reported in [206]. The introduced modifications can serve a dual role: They can act as potential sites for crack initiation or effectively redirect the growth of cracks that originate within the adhesive, particularly at points of high adhesive strain concentration. By impeding the propagation of adhesive cracks into the laminate, these modifications play a critical role in preventing damage to the overall composite adherends, consequently improving the fatigue strength of joints.

## 6. Design Guidelines

In this dedicated section, we provide a comprehensive summary of how various parameters impact the fatigue performance of adhesively bonded composite joints. The objective is to offer a concise yet informative design guideline that highlights the critical factors influencing the behavior of these joints under cyclic loading conditions. These guidelines are categorized into three main approaches: Configurations, adhesive modification, and adherend modification. The paper concludes by summarizing the key findings and insights from each category, highlighting the strategies employed to enhance the fatigue performance of composite adhesive joints. 

**Configuration**

- The utilization of a corrugated interface has resulted in a substantial enhancement of approximately 40% in static strength [68]. In terms of fatigue loading conditions, it was observed that the fatigue life of the material increased by up to five times, depending on the applied load level [46].
- The implementation of reverse bent joints has led to a substantial improvement of up to 190% in static strength compared to flat interface geometry. Additionally, in terms of fatigue performance, the increase in strength can be greater than what is observed in static conditions [64]. Nonetheless, achieving precise control over the geometry and manufacturing of curved composite adherends remains a significant challenge.
- Increases in overlap length have been shown to correspondingly increase the static failure load of the joint until the adherends reach a critical point [23,75]. However, this effect becomes negligible when utilizing a brittle and stiff adhesive [23]. To achieve optimal fatigue life and strength, it is advisable to determine a minimum overlap length [27]. In general, extending the overlap length tends to enhance the fatigue load [35,44,85]. Nevertheless, conflicting trends for the effect of the overlap length on fatigue strength have been reported in some references. These inconsistencies can be attributed to variations in the adhesive (Brittle or ductile) and composite adherend properties as well as the initiation failure mode.
- Applying a fillet to the adherend corner eliminates the singularity in that particular region [44,85]. Utilizing a fillet geometry has been shown to enhance both the static and fatigue strength of adhesively bonded composite joints [93]. This improvement can be attributed to the reduced intensity and degree of singularity associated with the fillet corner geometry compared to a sharp square edge [90]. Nevertheless, the extent of the fatigue strength increase achieved through the use of fillets may be influenced by other factors, including the overlap length and other geometric parameters of the joint. Consequently, it is essential to carefully assess and optimize these parameters in order to attain the desired fatigue performance.

**Adherend**

- Considering the adhesive/adherend interface layer orientation, it is important to note that it can have an impact on both the static strength and fatigue behavior of the joint. In terms of static strength, there is a possibility of a slight increase when specific orientations are employed [27,50,52,99,155]. The failure mode also varies depending on the interface orientation. In the case of a 0° interface, failure typically initiates due to adhesive/adherend interface debonding. However, when a 90° interface is present, failure tends to happen inside the composite adherends [130]. Furthermore, the orientation of the adhesive adjacent layers plays a role in fatigue crack propagation. With a 0° orientation, cracks predominantly propagate within the adhesive. However, at 45 and 90°, the crack growth becomes more complex. Using a 90° layer as the interface between adherends and adhesive represents the worst case in terms of fatigue strength, while the 0° orientation is expected to deliver the best performance [27,155,158].
- The stacking sequence has a significant impact on the static failure load simply by modifying the layup configuration. Notably, the majority of [0]_16_ plies demonstrated higher failure loads, except for the [0/45/−45/90]_4_ configuration, which exhibited 48% greater strength compared to the stacking sequence consisting entirely of 0° plies [23]. In terms of fatigue strength, the proportion of adherends’ layers oriented at 0° plays a crucial role in determining the joint’s resistance to fatigue [79,156]. It should be noted that the impact of these factors on fatigue strength varies significantly based on the joint configuration.
- The strength of the joint can be enhanced by using adherends with higher stiffness specifically for bending and peel resistance [152,153]. This can be achieved by either increasing the number of layers oriented at 0° or positioning them closer to the outer surfaces of the adherends [130]. Similar to the static failure behavior, the fracture characteristics under cyclic loading are influenced by the stress distribution along the bondline, which, in turn, is affected by the rigidity of the adherends [27]. In general, utilizing a stiffer laminate as adherends tends to improve the fatigue strength of the joint [79].
- In the context of surface treatment, the ultimate static strength of an adhesive-bonded joint is lower when the failure occurs at the adhesive layer as compared to a cohesive failure. The effectiveness of pretreatments has a notable impact on this aspect [43]. Furthermore, while failure is attributed to the adhesive, there is a considerable reduction in the fatigue resistance of the bonded joint. Consequently, employing an effective surface treatment can shift the failure mode toward cohesive failure, leading to an increase in fatigue strength [43].

**Adhesive**

- According to studies, the addition of nanoparticles may have either a deleterious or enhancing effect on static strength, depending on the type of nanoparticles used and the adhesive type [190,191,192,193].
- When it comes to the mixed adhesive layer, the improvement in static strength is not guaranteed and largely depends on the specific adhesive materials used, as well as the length ratio of the layers [199,207]. However, the effect of mixed adhesive layers on the fatigue strength of adhesively bonded composite joints is still relatively unexplored and should be addressed in future research.
- The impact of adhesive thickness on joint strength is contingent upon the ductility of the adhesive. In the case of brittle adhesives, there is a critical thickness beyond which strength begins to decline as the adhesive thickness increases. Conversely, for ductile adhesives, an increase in thickness can result in a reduction in static strength [95,96,106]. Regarding fatigue strength, studies have shown a decrease in fatigue resistance as the bondline thickness increases [50,98]. However, there exists a specific thickness at which the maximum fatigue resistance is observed [27].

## 7. Conclusions

The fatigue behavior of adhesively bonded composite joints has been reviewed, with a focus on enhancing their fatigue life and strength. The complexity of composite materials, the limited research in this area, and the presence of numerous effective parameters, including overlap length, bondline thickness, and joint configurations, along with their interdependencies, make it difficult to draw definitive conclusions.

- It has been shown that changing the geometry of joints can increase the fatigue strength. These changes can take place in adhesive thickness, overlap length, and shape. The influence of these parameters on fatigue life is interconnected, meaning that to investigate the effect of them on fatigue behavior, they should be assessed simultaneously.
- The primary factor that significantly influences joint strength is the configuration of the joints. While wavy, step, butt, and double lap joints have demonstrated superior fatigue strength compared to traditional single lap joints, it is worth noting that the scarf joint has exhibited the most significant enhancement in this regard.
- Increasing the overlap length had a more significant impact on improving the fatigue strength of adhesively bonded composite joints compared to the use of corner fillets.
- In the context of composite adherends and their stacking sequence, the proportion of 0° layers directly affects the rise in fatigue strength and also alters the mode of failure by affecting the adherends’ stiffness.
- Applying an effective surface treatment can shift the failure mode toward cohesive failure, leading to an increase in fatigue strength.
- Modification of the adhesive layer can be achieved through the utilization of either a mixed adhesive layer or an adhesive layer reinforced with nanomaterials. It has been demonstrated that adhesive modification can enhance the fatigue behavior of adhesively bonded composite joints, depending on the type of adhesive and nanoparticles used.

## Data Availability

Not applicable.

## References

[1] Maiti S., Islam M.R., Uddin M.A., Afroj S., Eichhorn S.J., Karim N. (2022). Sustainable Fiber-Reinforced Composites: A Review. Adv. Sustain. Syst..

[2] Bahabadi H.M., Farrokhabadi A., Rahimi G.H. (2020). Investigation of debonding growth between composite skins and corrugated foam-composite core in sandwich panels under bending loading. Eng. Fract. Mech..

[3] Compton B.G., Post B.K., Duty C.E., Love L., Kunc V. (2017). Thermal analysis of additive manufacturing of large-scale thermoplastic polymer composites. Addit. Manuf..

[4] Mohan V.R., Tamma K.K., Shires D.R., Mark A. (1998). Advanced manufacturing of large-scale composite structures: Process modeling, manufacturing simulations and massively parallel computing platforms. Adv. Eng. Softw..

[5] Wang C., Huang Y.D., Xv H.Y., Liu W.B. (2004). The durability of adhesive/carbon–carbon composites joints in salt water. Int. J. Adhes. Adhes..

[6] Borges C.S.P., Akhavan-Safar A., Tsokanas P., Carbas R.J.C., Marques E.A.S., da Silva L.F.M. (2023). From fundamental concepts to recent developments in the adhesive bonding technology: A general view. Discov. Mech. Eng..

[7] Budzik M.K., Wolfahrt M., Reis P., Kozłowski M., Sena-Cruz J., Papadakis L., Nasr Saleh M., Machalicka K.V., Teixeira de Freitas S., Vassilopoulos A.P. (2022). Testing mechanical performance of adhesively bonded composite joints in engineering applications: An overview. J. Adhes..

[8] Higgins A. (2000). Adhesive bonding of aircraft structures. Int. J. Adhes. Adhes..

[9] Qin Z., Yang K., Wang J., Zhang L., Huang J., Peng H., Xu J. (2021). The effects of geometrical dimensions on the failure of composite-to-composite adhesively bonded joints. J. Adhes..

[10] Dilthey U., Stein L. (2006). Multimaterial car body design: Challenge for welding and joining. Sci. Technol. Weld. Join..

[11] Di Bella G., Galtieri G., Pollicino E., Borsellino C. (2013). Mechanical characterization of adhesive joints with dissimilar substrates for marine applications. Int. J. Adhes. Adhes..

[12] Delzendehrooy F., Akhavan-Safar A., Barbosa A.Q., Beygi R., Cardoso D., Carbas R.J.C., Marques E.A.S., da Silva L.F.M. (2022). A comprehensive review on structural joining techniques in the marine industry. Compos. Struct..

[13] Hizam R.M., Manalo A.C., Karunasena W. (2013). A review of FRP composite truss systems and its connections. From Materials to Structures: Advancement through Innovation—Proceedings of the 22nd Australasian Conference on the Mechanics of Structures and Materials, ACMSM 22, Sydney, Australia, 11–14 December 2012.

[14] Budhe S., Banea M.D., de Barros S. (2018). Bonded repair of composite structures in aerospace application: A review on environmental issues. Appl. Adhes. Sci..

[15] Katnam K.B., Da Silva L.F.M., Young T.M. (2013). Bonded repair of composite aircraft structures: A review of scientific challenges and opportunities. Prog. Aerosp. Sci..

[16] Chester R.J., Walker K.F., Chalkley P.D. (1999). Adhesively bonded repairs to primary aircraft structure. Int. J. Adhes. Adhes..

[17] da Silva L.F.M., Öchsner A.A.R. (2018). Handbook of Adhesion Technology: Second Edition.

[18] Kim C.-H., Choi J.-H., Kweon J.-H. (2015). Defect detection in adhesive joints using the impedance method. Compos. Struct..

[19] Ariaee S., Tutunchi A., Kianvash A., Entezami A.A. (2014). Modeling and optimization of mechanical behavior of bonded composite–steel single lap joints by response surface methodology. Int. J. Adhes. Adhes..

[20] de Castro Lopes F.V.B., Akhavan-Safar A., Carbas R.J.C., Marque E.A.S., Goyal R., Jennings J., da Silva L.F.M. (2023). The role of loading mode on the property degradation of adhesives at high temperatures. Fatigue Fract. Eng. Mater. Struct..

[21] Shang X., Marques E.A.S., Machado J.J.M., Carbas R.J.C., Jiang D., da Silva L.F.M. (2019). Review on techniques to improve the strength of adhesive joints with composite adherends. Compos. Part B Eng..

[22] Ramalho L.D.C., Campilho R.D.S.G., Belinha J., da Silva L.F.M. (2020). Static strength prediction of adhesive joints: A review. Int. J. Adhes. Adhes..

[23] Ramezani F., Simões B.D., Carbas R.J.C., Marques E.A.S., da Silva L.F.M. (2023). Developments in Laminate Modification of Adhesively Bonded Composite Joints. Materials.

[24] (1999). Standard Practice for Classifying Failure Modes in Fiber-Reinforced Plastic (FRP).

[25] Khalili S.M.R., Shokuhfar A., Hoseini S.D., Bidkhori M., Khalili S., Mittal R.K. (2008). Experimental study of the influence of adhesive reinforcement in lap joints for composite structures subjected to mechanical loads. Int. J. Adhes. Adhes..

[26] Sharba M.J., Leman Z., Sultan M.T.H., Ishak M.R., Hanim M.A.A. (2015). Monotonic and fatigue properties of kenaf/glass hybrid composites under fully reversed cyclic loading. IOP Conf. Ser. Mater. Sci. Eng..

[27] De Goeij W.C., Van Tooren M.J.L., Beukers A. (1999). Composite adhesive joints under cyclic loading. Mater. Des..

[28] Zamani P., Jaamialahmadi A., da Silva L.F.M. (2023). Fatigue life evaluation of Al-GFRP bonded lap joints under four-point bending using strain-life criteria. Int. J. Adhes. Adhes..

[29] Beber V.C., Schneider B., Brede M. (2016). Influence of Temperature on the Fatigue Behaviour of a Toughened Epoxy Adhesive. J. Adhes..

[30] de Castro Lopes F.V.B., Akhavan-Safar A., Carbas R.J.C., Marques E.A.S., Goyal R., Jennings J., da Silva L.F.M. (2023). The interaction of loading mode and humidity on the properties degradation of an epoxy adhesive subjected to strength, fracture, and fatigue tests. J. Appl. Polym. Sci..

[31] de Barros S., Kenedi P.P., Ferreira S.M., Budhe S., Bernardino A.J., Souza L.F.G. (2017). Influence of mechanical surface treatment on fatigue life of bonded joints. J. Adhes..

[32] Javaid U., Ling C., Cardiff P. (2020). Mechanical performance of carbon-glass hybrid composite joints in quasi-static tension and tension-tension fatigue. Eng. Fail. Anal..

[33] Ramírez F.M.G., Garpelli F.P., de Sales R.C.M., Cândido G.M., Arbelo M.A., Shiino M.Y., Donadon M.V. (2020). Hygrothermal effects on the fatigue delamination growth onset in interlayer toughened CFRP joints. Int. J. Fatigue.

[34] Zamani P., Alaei M.H., da Silva L.F.M., Ghahremani-Moghadam D. (2022). On the static and fatigue life of nano-reinforced Al-GFRP bonded joints under different dispersion treatments. Fatigue Fract. Eng. Mater. Struct..

[35] Ishii K., Imanaka M., Nakayama H., Kodama H. (1998). Fatigue failure criterion of adhesively bonded CFRP/metal joints under multiaxial stress conditions. Compos. Part A Appl. Sci. Manuf..

[36] Khashaba U.A., Najjar I.M.R., Almitani K.H. (2022). Failure analysis of scarf adhesive joints modified with SiC-nanoparticles under fatigue loading at room temperature. Compos. Sci. Technol..

[37] Anderson T.L. (2016). Fracture Mechanics: Fundamentals and Applications Surjya Kumar Maiti. MRS Bull..

[38] Pedro P., Camanho S.R.H., Vassilopoulos A.P. (2015). Fatigue and Fracture of Adhesively-Bonded Composite Joints: Behaviour, Simulation and Modelling.

[39] Olajide S.O., Arhatari B.D. (2017). Recent progress on damage mechanisms in polymeric adhesively bonded high-performance composite joints under fatigue. Int. J. Fatigue.

[40] Augustin T., Karsten J., Kötter B., Fiedler B. (2018). Health monitoring of scarfed CFRP joints under cyclic loading via electrical resistance measurements using carbon nanotube modified adhesive films. Compos. Part A Appl. Sci. Manuf..

[41] Palanisamy R.P., Banerjee P., Mukherjee S., Haq M., Deng Y. Fatigue damage prognosis in adhesive bonded composite lap-joints using guided waves. Proceedings of the 2020 IEEE International Conference on Prognostics and Health Management (ICPHM).

[42] Carboni M., Bernasconi A. (2021). Acoustic Emission Based Monitoring of Fatigue Damage in CFRP-CFRP Adhesive Bonded Joints.

[43] Ourahmoune R., Salvia M., Mathia T.G. (2016). Fatigue life analysis of adhesively bonded CFR-PEEK composites using acoustic emission monitoring. Rev. Compos. Mater. Av..

[44] Quaresimin M., Ricotta M. (2006). Fatigue behaviour and damage evolution of single lap bonded joints in composite material. Compos. Sci. Technol..

[45] Ishii K., Imanaka M., Nakayama H., Kodama H. (1999). Evaluation of the fatigue strength of adhesively bonded CFRP/metal single and single-step double-lap joints. Compos. Sci. Technol..

[46] Zeng Q., Sun C.T. (2004). Fatigue performance of a bonded wavy composite lap joint. Fatigue Fract. Eng. Mater. Struct..

[47] Cheuk P.T., Tong L., Wang C.H., Baker A., Chalkley P. (2002). Fatigue crack growth in adhesively bonded composite-metal double-lap joints. Compos. Struct..

[48] Crocombe A.D., Ong C.Y., Chan C.M., Wahab M.M.A., Ashcroft I.A. (2002). Investigating Fatigue Damage Evolution In Adhesively Bonded Structures Using Backface Strain Measurement. J. Adhes..

[49] Graner Solana A., Crocombe A.D., Ashcroft I.A. (2010). Fatigue life and backface strain predictions in adhesively bonded joints. Int. J. Adhes. Adhes..

[50] Tang J.H., Sridhar I., Srikanth N. (2013). Static and fatigue failure analysis of adhesively bonded thick composite single lap joints. Compos. Sci. Technol..

[51] Rocha A.V.M., Akhavan-Safar A., Carbas R., Marques E.A.S., Goyal R., El-zein M., da Silva L.F.M. (2020). Fatigue crack growth analysis of different adhesive systems: Effects of mode mixity and load level. Fatigue Fract. Eng. Mater. Struct..

[52] Renton W.J., Vinson J.R. (1975). Fatigue behavior of bonded joints in composite material structures. J. Aircr..

[53] Moreira R.D.F., de Moura M.F.S.F., Silva F.G.A., Reis J.P. (2020). High-cycle fatigue analysis of adhesively bonded composite scarf repairs. Compos. Part B Eng..

[54] Olajide S.O., Kandare E., Khatibi A.A. (2017). Fatigue life uncertainty of adhesively bonded composite scarf joints–an airworthiness perspective. J. Adhes..

[55] Yoo J.S., Truong V.H., Park M.Y., Choi J.H., Kweon J.H. (2016). Parametric study on static and fatigue strength recovery of scarf-patch-repaired composite laminates. Compos. Struct..

[56] Kim J.H., Park B.J., Han Y.W. (2004). Evaluation of fatigue characteristics for adhesively-bonded composite stepped lap joint. Compos. Struct..

[57] Matthews F.L., Kilty P.F., Godwin E.W. (1982). A review of the strength of joints in fibre-reinforced plastics. Part 2. Adhesively bonded joints. Composites.

[58] Boss J.N., Ganesh V.K., Lim C.T. (2003). Modulus grading versus geometrical grading of composite adherends in single-lap bonded joints. Compos. Struct..

[59] Her S.-C. (1999). Stress analysis of adhesively-bonded lap joints. Compos. Struct..

[60] Razavi S.M.J., Ayatollahi M.R., Samari M., da Silva L.F.M. (2019). Effect of interface non-flatness on the fatigue behavior of adhesively bonded single lap joints. Proc. Inst. Mech. Eng. Part L J. Mater. Des. Appl..

[61] Akrami R., Anjum S., Fotouhi S., Boaretto J., de Camargo F.V., Fotouhi M. (2021). Investigating the effect of interface morphology in adhesively bonded composite wavy-lap joints. J. Compos. Sci..

[62] Daniel V., Pires C. (2023). Department of Mechanical Engineering Study of a Novel Curved Single Lap Joint Concept with Non-Uniform Adhesive Thickness. Master’s Thesis.

[63] McLaren A.S., MacInnes I. (1958). The influence on the stress distribution in an adhesive lap joint of bending of the adhering sheets. Br. J. Appl. Phys..

[64] Fessel G., Broughton J., Fellows N., Durodola J., Hutchinson A. (2007). A numerical and experimental study on reverse-bent joints for composite substrates. Proceedings of the 48th AIAA/ASME/ASCE/AHS/ASC Structures, Structural Dynamics, and Materials Conference.

[65] Campilho R.D.S.G., Pinto A.M.G., Banea M.D., Silva R.F., da Silva L.F.M. (2011). Strength Improvement of Adhesively-Bonded Joints Using a Reverse-Bent Geometry. J. Adhes. Sci. Technol..

[66] Bodepudi R., Hughes D.J., Pradhan A.K. (2017). Evaluation of optimum configuration of adhesively bonded reverse bent joints. Int. J. Adhes. Adhes..

[67] Fessel G., Broughton J.G., Fellows N.A., Durodola J.F., Hutchinson A.R. (2007). Evaluation of different lap-shear joint geometries for automotive applications. Int. J. Adhes. Adhes..

[68] Ávila A.F., de Bueno P.O. (2004). Stress analysis on a wavy-lap bonded joint for composites. Int. J. Adhes. Adhes..

[69] Ashrafi M., Ajdari A., Rahbar N., Papadopoulos J., Nayeb-Hashemi H., Vaziri A. (2012). Adhesively bonded single lap joints with non-flat interfaces. Int. J. Adhes. Adhes..

[70] Ayatollahi M.R., Samari M., Razavi S.M.J., da Silva L.F.M. (2017). Fatigue performance of adhesively bonded single lap joints with non-flat sinusoid interfaces. Fatigue Fract. Eng. Mater. Struct..

[71] Zeng Q., Sun C.T. Fatigue performance of a bonded wavy composite lap joint. Proceedings of the 19th AIAA Applied Aerodynamics Conference.

[72] Zeng Q., Sun C. (2000). A new bonded composite wavy lap joint. 41st Structures, Structural Dynamics, and Materials Conference and Exhibit, Atalanta, GA, USA, 3–6 April 2000.

[73] Zeng Q.-G., Sun C.T. (2001). Novel Design of a Bonded Lap Joint. AIAA J..

[74] Sharma R., Gupta A. (2023). A critical review on influencing parameters for adhesively bonded joints in composite laminates for structural applications. Mater. Today Proc..

[75] Li J., Yan Y., Zhang T., Liang Z. (2015). Experimental study of adhesively bonded CFRP joints subjected to tensile loads. Int. J. Adhes. Adhes..

[76] Canyurt O.E., Meran C., Uslu M. (2010). Strength estimation of adhesively bonded tongue and groove joint of thick composite sandwich structures using genetic algorithm approach. Int. J. Adhes. Adhes..

[77] Canyurt O.E., Meran C. (2012). Fatigue strength estimation of adhesively bonded tongue and groove joint of thick woven composite sandwich structures using genetic algorithm approach. Int. J. Adhes. Adhes..

[78] Kupski J., Teixeira de Freitas S. (2021). Design of adhesively bonded lap joints with laminated CFRP adherends: Review, challenges and new opportunities for aerospace structures. Compos. Struct..

[79] Ferreira J.A.M., Reis P.N., Costa J.D.M., Richardson M.O.W. (2002). Fatigue behaviour of composite adhesive lap joints. Compos. Sci. Technol..

[80] Kang T.Y., Aan H.S., Chun H.J., Park J.C. (2023). Fatigue and Tensile Behaviors of High Stiffness Adhesive Bonded and Hybrid Joints with Composite-Steel Dissimilar Materials. Int. J. Precis. Eng. Manuf..

[81] Osiyemi S.O. (1992). The Fatigue Performance of Adhesively Bonded Fibre-Composite Joints. Ph.D. Thesis.

[82] Kara E., KurŞun A., Haboğlu M.R., Enginsoy H.M., Aykul H. (2015). Fatigue behavior of adhesively bonded glass fiber reinforced plastic composites with different overlap lengths. Proc. Inst. Mech. Eng. Part C J. Mech. Eng. Sci..

[83] Meneghetti G., Quaresimin M., Ricotta M. (2012). Damage mechanisms in composite bonded joints under fatigue loading. Compos. Part B Eng..

[84] Da Costa Mattos H.S., Monteiro A.H., Palazzetti R. (2012). Failure analysis of adhesively bonded joints in composite materials. Mater. Des..

[85] Quaresimin M., Ricotta M. (2006). Life prediction of bonded joints in composite materials. Int. J. Fatigue.

[86] Mall S., Ramamurthy G. (1989). Effect of bond thickness on fracture and fatigue strength of adhesively bonded composite joints. Int. J. Adhes. Adhes..

[87] Lang T.P., Mallick P.K. (1998). Effect of spew geometry on stresses in single lap adhesive joints. Int. J. Adhes. Adhes..

[88] da Silva L.F.M., Adams R.D. (2007). Joint strength predictions for adhesive joints to be used over a wide temperature range. Int. J. Adhes. Adhes..

[89] da Silva L.F.M., Marques E.A.S., Campilho R.D.S.G., da Silva L.F.M., Öchsner A., Adams R.D. (2018). Design Rules and Methods to Improve Joint Strength BT—Handbook of Adhesion Technology.

[90] Quaresimin M., Ricotta M. (2006). Stress intensity factors and strain energy release rates in single lap bonded joints in composite materials. Compos. Sci. Technol..

[91] Crocombe A.D. (1989). Global yielding as a failure criterion for bonded joints. Int. J. Adhes. Adhes..

[92] Hart-Smith L.J. (1985). Designing to Minimize Peel Stresses in Adhesive-Bonded Joints.

[93] Niranjan V. (1970). Bonded Joints: A Review for Engineers.

[94] da Silva L.F.M., Rodrigues T.N.S.S., Figueiredo M.A.V., de Moura M.F.S.F., Chousal J.A.G. (2006). Effect of Adhesive Type and Thickness on the Lap Shear Strength. J. Adhes..

[95] Taib A.A., Boukhili R., Achiou S., Gordon S., Boukehili H. (2006). Bonded joints with composite adherends. Part I. Effect of specimen configuration, adhesive thickness, spew fillet and adherend stiffness on fracture. Int. J. Adhes. Adhes..

[96] Diharjo K., Anwar M., Tarigan R.A.P., Rivai A. (2016). Effect of adhesive thickness and surface treatment on shear strength on single lap joint Al/CFRP using adhesive of epoxy/Al fine powder. AIP Conf. Proc..

[97] Mazumdar S.K., Mallick P.K. (1998). Static and fatigue behavior of adhesive joints in SMC-SMC composites. Polym. Compos..

[98] Sekiguchi Y., Sato C. (2021). Effect of bond-line thickness on fatigue crack growth of structural acrylic adhesive joints. Materials.

[99] Renton W.J., Vinson J.R. (1975). The Efficient Design of Adhesive Bonded Joints. J. Adhes..

[100] Ebadi-Rajoli J., Akhavan-Safar A., Hosseini-Toudeshky H., da Silva L.F.M. (2020). Progressive damage modeling of composite materials subjected to mixed mode cyclic loading using cohesive zone model. Mech. Mater..

[101] Crocombe A.D., Moult A.C., Allen K.W. (1988). The Effect of the Adhesive Thickness on the Strength of a Bonded Joint BT—Adhesion 12.

[102] Findley W.N., Worley W.J. (1948). Mechanical Properties of Five Laminated Plastics.

[103] Boller K.H. (1952). Fatigue Tests of Glass-Fabric-Base Laminates Subjected to Axial Loading.

[104] Boller K.H. (1957). Fatigue properties of fibrous glass-reinforced plastics laminates subjected to various conditions. Mod. Plast..

[105] Hashim N., Majid D.L.A., Mahdi E.-S., Zahari R., Yidris N. (2019). Effect of fiber loading directions on the low cycle fatigue of intraply carbon-Kevlar reinforced epoxy hybrid composites. Compos. Struct..

[106] Dai G., Mishnaevsky L. (2014). Fatigue of hybrid glass/carbon composites: 3D computational studies. Compos. Sci. Technol..

[107] Shahzad A. (2011). Impact and fatigue properties of hemp–glass fiber hybrid biocomposites. J. Reinf. Plast. Compos..

[108] Salkind M.J. (1972). Fatigue of composites. Composite Materials, Testing and Design, Proceedings of the 2nd Conference.

[109] Malekinejadbahabadi H., Farrokhabadi A., Rahimi G.H., Nazerigivi A. (2022). Effect of core shape on debonding failure of composite sandwich panels with foam-filled corrugated core. Steel Compos. Struct..

[110] Berthelot J.-M. (2003). Transverse cracking and delamination in cross-ply glass-fiber and carbon-fiber reinforced plastic laminates: Static and fatigue loading. Appl. Mech. Rev..

[111] Vassilopoulos A.P. (2020). The history of fiber-reinforced polymer composite laminate fatigue. Int. J. Fatigue.

[112] Amraei J., Katunin A. (2022). Recent Advances in Limiting Fatigue Damage Accumulation Induced by Self-Heating in Polymer&ndash;Matrix Composites. Polymers.

[113] Talreja R. (1981). Fatigue of composite materials: Damage mechanisms and fatigue-life diagrams. Proc. R. Soc. Lond. A. Math. Phys. Sci..

[114] Alam P., Mamalis D., Robert C., Floreani C., Brádaigh C.M.Ó. (2019). The fatigue of carbon fi bre reinforced plastics—A review. Compos. Part B.

[115] Jones C.J., Dickson R.F., Adam T., Reiter H., Harris B. (1984). The environmental fatigue behaviour of reinforced plastics. Proc. Math. Phys. Eng. Sci..

[116] Konur O., Matthews F.L. (1989). Effect of the properties of the constituents on the fatigue performance of composites: A review. Composites.

[117] Curtis P.T., Moore B.B., Marshall I.H. (1983). A Comparison of Plain and Double Waisted Coupons for Static and Fatigue Tensile Testing of Unidirectional GRP and CFRP BT—Composite Structures 2.

[118] Hull D.C.T. (1998). An Introduction to Composite Materials.

[119] Sabiston T., Li B., Muhammad W., Kang J., Engler-Pinto C. (2022). The Role of Fibre Length on the Fatigue Failure of Injection-Moulded Composites at Elevated Temperatures under a Range of Axial Loading Conditions. J. Compos. Sci..

[120] Karger-Kocsis J., Friedrich K. (1988). Fatigue crack propagation in short and long fibre-reinforced injection-moulded PA 6.6 composites. Composites.

[121] Meneghetti G., Ricotta M., Lucchetta G., Carmignato S. (2014). An hysteresis energy-based synthesis of fully reversed axial fatigue behaviour of different polypropylene composites. Compos. Part B Eng..

[122] Naik P.S., Orangalu S.A., Londhe N. (2012). V Effect of fiber weight fraction on mechanical properties of carbon–carbon composites. Polym. Compos..

[123] Brunbauer J., Pinter G. (2015). Effects of mean stress and fibre volume content on the fatigue-induced damage mechanisms in CFRP. Int. J. Fatigue.

[124] Hiremath C., Senthilnathan K., Guha A., Tewari A. (2015). Effect of Volume Fraction on Damage Accumulation for a Lattice Arrangement of Fibers in CFRP. Mater. Today Proc..

[125] Brunbauer J., Stadler H., Pinter G. (2015). Mechanical properties, fatigue damage and microstructure of carbon/epoxy laminates depending on fibre volume content. Int. J. Fatigue.

[126] Roundi W., El Mahi A., El Gharad A., Rebière J.-L. (2017). Experimental and numerical investigation of the effects of stacking sequence and stress ratio on fatigue damage of glass/epoxy composites. Compos. Part B Eng..

[127] Mandell J.F., Damborsky D.D. (1997). SNL/MSU/DOE Composite Materials Fatigue Database V22.0.

[128] Sharma N., Singh K.K. (2023). Transverse fatigue behavior analysis of symmetric and asymmetric glass fiber-reinforced laminates. Polym. Compos..

[129] Şen I., Alderliesten R.C., Benedictus R. (2015). Lay-up optimisation of fibre metal laminates based on fatigue crack propagation and residual strength. Compos. Struct..

[130] Kupski J., Teixeira de Freitas S., Zarouchas D., Camanho P.P., Benedictus R. (2019). Composite layup effect on the failure mechanism of single lap bonded joints. Compos. Struct..

[131] Maa R.-H., Cheng J.-H. (2002). A CDM-based failure model for predicting strength of notched composite laminates. Compos. Part B Eng..

[132] Zhao X., Wang X., Wu Z., Keller T., Vassilopoulos A.P. (2019). Temperature effect on fatigue behavior of basalt fiber-reinforced polymer composites. Polym. Compos..

[133] Movahedi-Rad A.V., Keller T., Vassilopoulos A.P. (2018). Fatigue damage in angle-ply GFRP laminates under tension-tension fatigue. Int. J. Fatigue.

[134] Turon A., Costa J., Camanho P.P., Dávila C.G. (2007). Simulation of delamination in composites under high-cycle fatigue. Compos. Part A Appl. Sci. Manuf..

[135] Ribeiro F., Sena-Cruz J., Vassilopoulos A.P. (2021). Tension-tension fatigue behavior of hybrid glass/carbon and carbon/carbon composites. Int. J. Fatigue.

[136] Zuo P., Srinivasan D.V., Vassilopoulos A.P. (2021). Review of hybrid composites fatigue. Compos. Struct..

[137] Tabrizi I.E., Kefal A., Zanjani J.S.M., Akalin C., Yildiz M. (2019). Experimental and numerical investigation on fracture behavior of glass/carbon fiber hybrid composites using acoustic emission method and refined zigzag theory. Compos. Struct..

[138] Wan Y.Z., Huang Y., He F., Li Q.Y., Lian J.J. (2007). Tribological properties of three-dimensional braided carbon/Kevlar/epoxy hybrid composites under dry and lubricated conditions. Mater. Sci. Eng. A Struct. Mater. Prop. Microstruct. Process..

[139] Chinnasamy V., Pavayee Subramani S., Palaniappan S.K., Mylsamy B., Aruchamy K. (2020). Characterization on thermal properties of glass fiber and kevlar fiber with modified epoxy hybrid composites. J. Mater. Res. Technol..

[140] Venkateshwaran N., Elayaperumal A., Sathiya G.K. (2012). Prediction of tensile properties of hybrid-natural fiber composites. Compos. Part B Eng..

[141] Dickson R.F., Fernando G., Adam T., Reiter H., Harris B. (1989). Fatigue behaviour of hybrid composites—Part 2 Carbon-glass hybrids. J. Mater. Sci..

[142] Hofer K.E., Stander M., Bennett L.C. (1978). Degradation and enhancement of the fatigue behavior of glass/graphite/epoxy hybrid composites after accelerated aging. Polym. Eng. Sci..

[143] Shan Y., Liao K. (2002). Environmental fatigue behavior and life prediction of unidirectional glass–carbon/epoxy hybrid composites. Int. J. Fatigue.

[144] Yu H., Longana M.L., Jalalvand M., Wisnom M.R., Potter K.D. (2015). Pseudo-ductility in intermingled carbon/glass hybrid composites with highly aligned discontinuous fibres. Compos. Part A Appl. Sci. Manuf..

[145] París F., Velasco M.L., Correa E., Van Paepegem W. (2021). 9—Modelling fibre–matrix interface debonding and matrix cracking in composite laminates. Multi-Scale Continuum Mechanics Modelling of Fibre-Reinforced Polymer Composites.

[146] Parvizi A., Garrett K.W., Bailey J.E. (1978). Constrained cracking in glass fibre-reinforced epoxy cross-ply laminates. J. Mater. Sci..

[147] Flaggs D.L., Kural M.H. (1982). Experimental Determination of the In Situ Transverse Lamina Strength in Graphite/Epoxy Laminates. J. Compos. Mater..

[148] Sánchez S. (2023). Damage and Failure Mechanisms under Fatigue in Long Fibre Composites with Ultra-Thin Plies, 2023. Ph.D. Thesis.

[149] Sánchez-Carmona S., Correa E., Barroso A., París F. (2023). Fatigue life of unidirectional 90° carbon/epoxy laminates made of conventional and ultra-thin plies varying manufacturing and testing conditions. Fatigue Fract. Eng. Mater. Struct..

[150] París F., Velasco M.L., Correa E. (2021). The scale effect in composites: An explanation physically based on the different mechanisms of damage involved in failure. Compos. Struct..

[151] Fuller J.D., Wisnom M.R. (2015). Exploration of the potential for pseudo-ductility in thin ply CFRP angle-ply laminates via an analytical method. Compos. Sci. Technol..

[152] Matthews F.L., Tester T.T. (1985). The influence of stacking sequence on the strength of bonded CFRP single lap joints. Int. J. Adhes. Adhes..

[153] Kairouz K.C., Matthews F.L. (1993). Strength and failure modes of bonded single lap joints between cross-ply adherends. Composites.

[154] Renton J., Vinson R. (1973). The Analysis and Design of Composite Material Bonded Joints under Static and Fatigue Loadings.

[155] Purimpat S., Jérôme R., Shahram A. (2013). Effect of fiber angle orientation on a laminated composite single-lap adhesive joint. Adv. Compos. Mater..

[156] Apalak Z.G., Apalak M.K., Genc M.S. (2006). Progressive damage modeling of an adhesively bonded unidirectional composite single-lap joint in tension at the mesoscale level. J. Thermoplast. Compos. Mater..

[157] Shin K.C., Lee J.J. (2002). Fatigue characteristics of a co-cured single lap joint subjected to cyclic tensile loads. J. Adhes. Sci. Technol..

[158] Johnson W.S., Mall S. (1986). Influence of interface ply orientation on fatigue damage of adhesively bonded composite joints. J. Compos. Technol. Res..

[159] Mall S., Johnson W.S., Everett R.A., Mittal K.L. (1984). Cyclic Debonding of Adhesively Bonded Composites BT—Adhesive Joints: Formation, Characteristics, and Testing.

[160] Casas-Rodriguez J.P., Ashcroft I.A., Silberschmidt V.V. (2008). Damage in adhesively bonded CFRP joints: Sinusoidal and impact-fatigue. Compos. Sci. Technol..

[161] Köckritz T., Schiefer T., Jansen I., Beyer E. (2013). Improving the bond strength at hybrid-yarn textile thermoplastic composites for high-technology applications by laser radiation. Int. J. Adhes. Adhes..

[162] Mittal K.L., Mittal K.L. (2004). Polymer Surface Modification: Relevance to Adhesion.

[163] Rhee K.Y., Yang J.-H. (2003). A study on the peel and shear strength of aluminum/CFRP composites surface-treated by plasma and ion assisted reaction method. Compos. Sci. Technol..

[164] Schmutzler H., Popp J., Büchter E., Wittich H., Schulte K., Fiedler B. (2014). Improvement of bonding strength of scarf-bonded carbon fibre/epoxy laminates by Nd:YAG laser surface activation. Compos. Part A Appl. Sci. Manuf..

[165] Wingfield J.R.J. (1993). Treatment of composite surfaces for adhesive bonding. Int. J. Adhes. Adhes..

[166] Bernardi C., Toury B., Salvia M., Contraires E., Dubreuil F., Virelizier F., Ourahmoune R., Surowiec B., Benayoun S. (2022). Effects of flaming on polypropylene long glass fiber composites for automotive bonding applications with polyurethane. Int. J. Adhes. Adhes..

[167] Pijpers A.P., Meier R.J. (2001). Adhesion behaviour of polypropylenes after flame treatment determined by XPS(ESCA) spectral analysis. J. Electron Spectros. Relat. Phenomena.

[168] Baldan A. (2012). Adhesion phenomena in bonded joints. Int. J. Adhes. Adhes..

[169] Islam M.S., Tong L., Falzon P.J. (2014). Influence of metal surface preparation on its surface profile, contact angle, surface energy and adhesion with glass fibre prepreg. Int. J. Adhes. Adhes..

[170] Kanerva M., Saarela O. (2013). The Peel Ply Surface Treatment for Adhesive Bonding of Composites: A Review. Int. J. Adhes. Adhes..

[171] Prolongo S.G., Gude M.R., Del Rosario G., Ureña A. (2010). Surface Pretreatments for Composite Joints: Study of Surface Profile by SEM Image Analysis. J. Adhes. Sci. Technol..

[172] Ramaswamy K., O’Higgins R.M., Kadiyala A.K., McCarthy M.A., McCarthy C.T. (2020). Evaluation of grit-blasting as a pre-treatment for carbon-fibre thermoplastic composite to aluminium bonded joints tested at static and dynamic loading rates. Compos. Part B Eng..

[173] Thull D., Zimmer F., Hofmann T., Holtmannspoetter J., Koerwien T., Hoffmann M. (2019). Investigation of fluorine-based release agents for structural adhesive bonding of carbon fibre reinforced plastics. Appl. Adhes. Sci..

[174] Park S.M., Roy R., Kweon J.H., Nam Y. (2020). Strength and failure modes of surface treated CFRP secondary bonded single-lap joints in static and fatigue tensile loading regimes. Compos. Part A Appl. Sci. Manuf..

[175] Sancaktar E., Gomatam R. (1998). A Study on the Effects of Surface Roughness on the Strength of Single Lap Joints.

[176] Banea M.D., Da Silva L.F.M. (2009). Adhesively bonded joints in composite materials: An overview. Proc. Inst. Mech. Eng. Part L J. Mater. Des. Appl..

[177] Johnson R.T., Blohowiak K., Osborne J., Wilson R., Flinn B.D. (2017). Improving Adhesive Bonding of Composites through Surface Characterization Using Inverse Gas Chromatography (IGC) Methods.

[178] Yang G., Yang T., Yuan W., Du Y. (2019). The influence of surface treatment on the tensile properties of carbon fiber-reinforced epoxy composites-bonded joints. Compos. Part B Eng..

[179] Thäsler T., Holtmannspötter J., Gudladt H.J. (2019). Surface topography influences on the fatigue behavior of composite joints. Key Eng. Mater..

[180] Liu X., Zheng G., Luo Q., Li Q., Sun G. (2021). Fatigue behavior of carbon fibre reinforced plastic and aluminum single-lap adhesive joints after the transverse pre-impact. Int. J. Fatigue.

[181] Naat N., Boutar Y., Naïmi S., Mezlini S., Da Silva L.F.M. (2023). Effect of surface texture on the mechanical performance of bonded joints: A review. J. Adhes..

[182] Zecchini M. (2018). Finite Element Stress Analysis on Non-Flat Adhesively Bonded Single Lap Joints with Sinusoid Interfaces. Master’s Thesis.

[183] Monteiro J., Salgado R.M., Da Rocha T., Pereira G., Marques E.A.S., Carbas R.J.C., Da Silva L.F.M. (2021). Effect of adhesive type and overlap length on the mechanical resistance of a simple overlap adhesive joint. U. Porto J. Eng..

[184] Davis M., Bond D. (1999). Principles and practices of adhesive bonded structural joints and repairs. Int. J. Adhes. Adhes..

[185] Marques D., Ribeiro M.L., Tita V. (2021). Comparative study of adhesive fatigue in aeronautical bonded joints: A numerical approach in the frequency domain. Proc. Inst. Mech. Eng. Part L J. Mater. Des. Appl..

[186] Lou S., Cheng B., Ren G., Li Y., Bai X., Chen P. (2023). Effect of adhesive modification and surface treatment of laminates on the single lap bonding joint properties of carbon fibre composites. J. Adhes..

[187] Akpinar I.A., Gültekin K., Akpinar S., Akbulut H., Ozel A. (2017). Experimental analysis on the single-lap joints bonded by a nanocomposite adhesives which obtained by adding nanostructures. Compos. Part B Eng..

[188] Gude M.R., Prolongo S.G., Gómez-del Río T., Ureña A. (2011). Mode-I adhesive fracture energy of carbon fibre composite joints with nanoreinforced epoxy adhesives. Int. J. Adhes. Adhes..

[189] Tutunchi A., Kamali R., Kianvash A. (2015). Adhesive strength of steel–epoxy composite joints bonded with structural acrylic adhesives filled with silica nanoparticles. J. Adhes. Sci. Technol..

[190] Kang M.-H., Choi J.-H., Kweon J.-H. (2014). Fatigue life evaluation and crack detection of the adhesive joint with carbon nanotubes. Compos. Struct..

[191] Budhe S., Banea M.D., de Barros S., da Silva L.F.M. (2017). An updated review of adhesively bonded joints in composite materials. Int. J. Adhes. Adhes..

[192] Park S.W., Kim B.C., Lee D.G. (2009). Tensile Strength of Joints Bonded With a Nano-particle-Reinforced Adhesive. J. Adhes. Sci. Technol..

[193] Omairey S., Jayasree N., Kazilas M. (2021). Defects and uncertainties of adhesively bonded composite joints. SN Appl. Sci..

[194] Zamani P., Jaamialahmadi A., da Silva L.F.M. (2021). The influence of GNP and nano-silica additives on fatigue life and crack initiation phase of Al-GFRP bonded lap joints subjected to four-point bending. Compos. Part B Eng..

[195] Bali G., Topkaya T. (2023). Effect of graphene nano-particle reinforcement on the fatigue behavior of adhesively bonded single lap joints. Int. J. Adhes. Adhes..

[196] Koricho E.G., Khomenko A., Haq M., Carroll J., Daly S. (2015). Fatigue Behavior of Glass-Bubbles Modified Adhesively Bonded Composite Joints BT—Fracture, Fatigue, Failure, and Damage Evolution.

[197] Khashaba U.A., Aljinaidi A.A., Hamed M.A. (2017). Fatigue and reliability analysis of nano-modified scarf adhesive joints in carbon fiber composites. Compos. Part B.

[198] Gültekin K., Akpinar S., Gürses A., Eroglu Z., Cam S., Akbulut H., Keskin Z., Ozel A. (2016). The effects of graphene nanostructure reinforcement on the adhesive method and the graphene reinforcement ratio on the failure load in adhesively bonded joints. Compos. Part B Eng..

[199] Machado J.J.M., Gamarra P.-R., Marques E.A.S., da Silva L.F.M. (2018). Numerical study of the behaviour of composite mixed adhesive joints under impact strength for the automotive industry. Compos. Struct..

[200] Abdel Wahab M.M. (2012). Fatigue in Adhesively Bonded Joints: A Review. ISRN Mater. Sci..

[201] Jairaja R., Naik G.N. (2019). Single and dual adhesive bond strength analysis of single lap joint between dissimilar adherends. Int. J. Adhes. Adhes..

[202] Jalali S., Ayatollahi M.R., Akhavan-Safar A., da Silva L.F.M. (2021). Effects of impact fatigue on residual static strength of adhesively bonded joints. Proc. Inst. Mech. Eng. Part L J. Mater. Des. Appl..

[203] Akhavan-Safar A., Eisaabadi Bozchaloei G., Jalali S., Beygi R., Ayatollahi M.R., da Silva L.F.M. (2023). Impact Fatigue Life of Adhesively Bonded Composite-Steel Joints Enhanced with the Bi-Adhesive Technique. Materials.

[204] Akhavan-Safar A., Eisaabadi B.G., Jalali S., Beygi R., da Silva L.F.M. (2022). Impact fatigue life improvement of bonded structures using the bi-adhesive technique. Fatigue Fract. Eng. Mater. Struct..

[205] Potter K.D., Guild F.J., Harvey H.J., Wisnom M.R., Adams R.D. (2001). Understanding and control of adhesive crack propagation in bonded joints between carbon fibre composite adherends I. Experimental. Int. J. Adhes. Adhes..

[206] Guild F.J., Potter K.D., Heinrich J., Adams R.D., Winsom M.R. (2001). Understanding and control of adhesive crack propagation in bonded joints between carbon fibre composite adherends II. Finite element analysis. Int. J. Adhes. Adhes..

[207] Machado J.J.M., Gamarra P.M.-R., Marques E.A.S., da Silva L.F.M. (2018). Improvement in impact strength of composite joints for the automotive industry. Compos. Part B Eng..

